# DDR2 controls the epithelial-mesenchymal-transition-related gene expression via c-Myb acetylation upon matrix stiffening

**DOI:** 10.1038/s41598-017-07126-7

**Published:** 2017-07-28

**Authors:** Daehwan Kim, Eunae You, Jangho Jeong, Panseon Ko, Jung-Woong Kim, Sangmyung Rhee

**Affiliations:** Department of Life Science, Chung-Ang University, Seoul, 06974 Republic of Korea

## Abstract

Increasing matrix stiffness caused by the extracellular matrix (ECM) deposition surrounding cancer cells is accompanied by epithelial–mesenchymal transition (EMT). Here, we show that expression levels of EMT marker genes along with discoidin domain receptor 2 (DDR2) can increase upon matrix stiffening. DDR2 silencing by short hairpin RNA downregulated EMT markers. Promoter analysis and chromatin immunoprecipitation revealed that c-Myb and LEF1 may be responsible for DDR2 induction during cell culture on a stiff matrix. Mechanistically, c-Myb acetylation by p300, which is upregulated on the stiff matrix, seems to be necessary for the c-Myb-and-LEF1–mediated DDR2 expression. Finally, we found that the c-Myb–DDR2 axis is crucial for lung cancer cell line proliferation and expression of EMT marker genes in a stiff environment. Thus, our results suggest that DDR2 regulation by p300 expression and/or c-Myb acetylation upon matrix stiffening may be necessary for regulation of EMT and invasiveness of lung cancer cells.

## Introduction

Fibrosis is related to fibroblast activation and mechanical remodelling of the extracellular matrix (ECM), resulting in tissue architecture destruction and increased matrix stiffness^1, 2^. ECM rigidity increased by fibrosis in tumour tissue has been found to be linked to pathological conditions including cancer cell proliferation and epithelial–mesenchymal transition (EMT)^2, 3^. In the case of pulmonary fibrosis in the lung tissue, for example, is major causes of lung cancer initiation^4^. A stiffened ECM in the tumour microenvironment confers elongated morphology onto the cells and promotes motility accompanied by expression of proliferation- and EMT-related factors such as Snail, Twist, and N-cadherin^2, 5–7^. Conversely, EMT involves promotion of tissue fibrosis, which reciprocally ensures a permissive microenvironment for cancer cell delamination from primary tumours and invasion through the ECM^7^. During invasion and metastasis, encounters between cancer cells and ECM components such as collagen are inevitable; in this regard, collagen receptors are important for cancer progression^8, 9^.

Discoidin domain receptors (DDRs), DDR1 and DDR2, are widely expressed collagen receptors^8, 10, 11^. Similar to other receptor tyrosine kinases, DDRs undergo receptor autophosphorylation after collagen binding, but this process is known to be unusually slow and involves prolonged stimulation^11^. DDRs consist of an extracellular region containing the discoidin domain, a transmembrane region connected to a cytoplasmic domain including an intracellular juxtamembrane region, and a catalytic domain. We have previously reported that a substantially longer intracellular juxtamembrane region of DDR2 plays a crucial role in DDR2 activation and lung cancer progression^12^. Because collagen is known to stimulate EMT^1, 13, 14^, DDR2 function in EMT during cancer progression cannot be ignored. Indeed, DDR2 contributes to metastasis of breast cancer cells that have undergone EMT via maintenance of SNAIL1 stability and activity^9^. DDR2 expression is also reported to mediate TGF-β–induced EMT in renal epithelial cells and lung cancers^14^. Nevertheless, the mechanisms controlling DDR2 expression during EMT are not clear.

In this study, we show that DDR2 expression accompanied with expression of EMT marker genes can increase during cell culture on a stiff matrix. Mechanistically, histone acetyltransferase (HAT) p300 upregulation on a stiff ECM increases the transcription factor c-Myb acetylation; this process appears to induce c-Myb binding along with LEF1 to the *DDR2* promoter, resulting in DDR2 upregulation in a stiff environment. Finally, we show that c-Myb silencing may lead to DDR2 inhibition and invasion by lung cancer cells; these effects are reversed by ectopic expression of DDR2. Therefore, our results suggest that DDR2 upregulated by matrix stiffening can play a critical role in EMT marker expression and invasiveness.

## Results

### ECM stiffness affects cellular behaviour and gene expression levels

**Figure 1:**
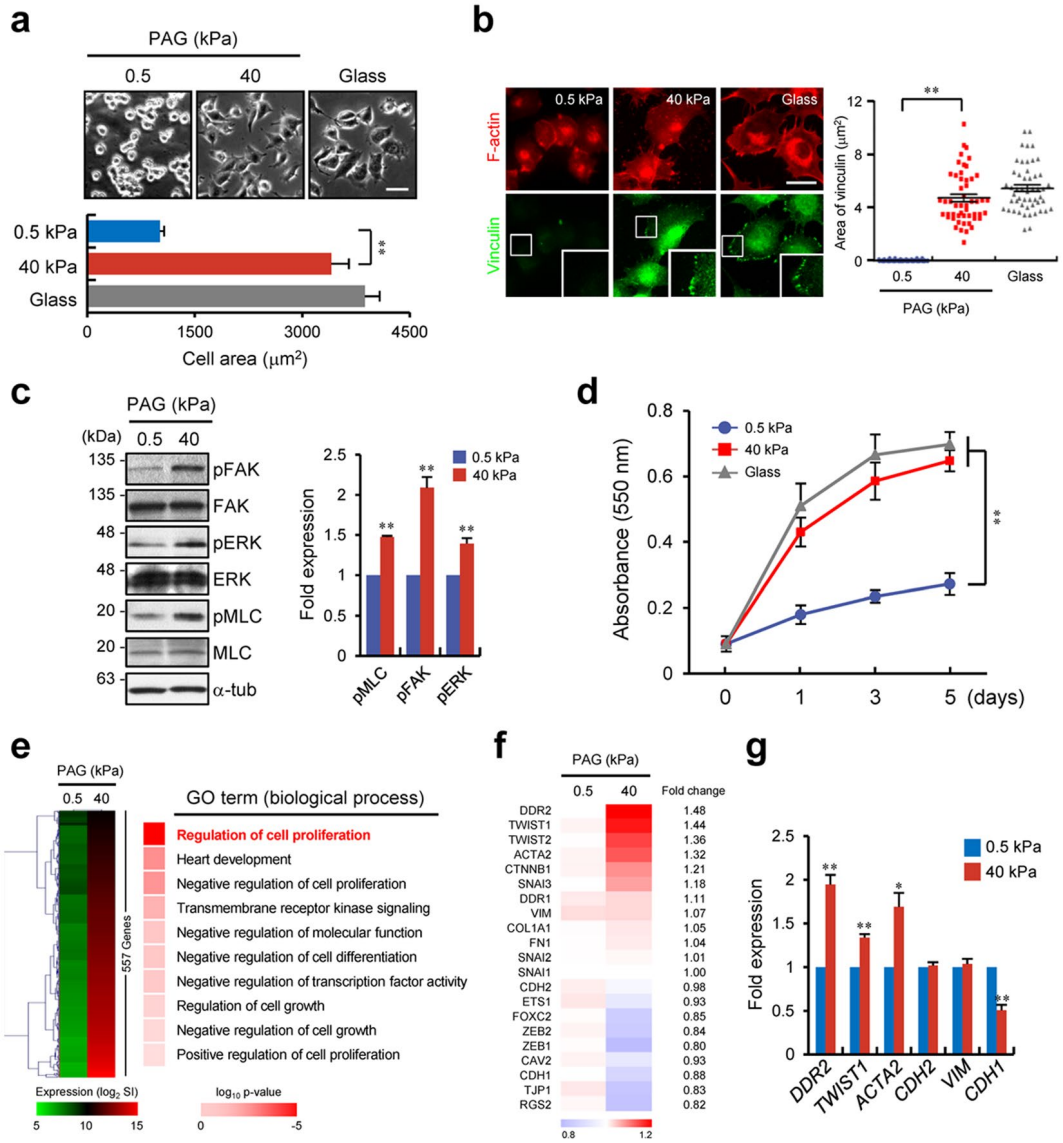
Stiffness of the ECM affects cellular behaviour and gene expression levels. (**a**) Phase contrast images of H1299 cells incubated on PAGs with the indicated stiffness or on glass. Scale bars, 50 μm. (**b**) Immunostaining of F-actin (red) and vinculin (green) in H1299 cells incubated under the indicated conditions (left); quantification of vinculin (n = 50) area for each condition (right). (**c**) Western blot analysis of pFAK, FAK, pERK, ERK, pMLC, and MLC in H1299 cells incubated under the indicated conditions. (**d**) Quantification of proliferation of H1299 cells incubated on a soft or stiff PAG with the indicated stiffness or on glass for the indicated periods. (**e**) Microarray gene expression analysis of H1299 cells incubated under the indicated conditions for 24 h. Heat map and gene ontology (GO) analysis of gene expression profiles of 557 upregulated genes with a ≥1.4× change in expression. (**f**) Heat map representation of microarray data showing the expression levels of several key EMT-related genes. (**g**) Quantitative RT-PCR (qRT-PCR) analysis of expression of EMT-related genes in H1299 cells incubated on a PAG with 0.5 or 40 kPa stiffness. qRT-PCR values were normalised to *GAPDH*. Data in (**a**), (**b**), (**c**), (**d**) and (**g**) represent the mean of three independent experiments ± SEM. ^*^ *P* < 0.05, ^**^ *P* < 0.01.

ECM stiffness is reported to affect cellular functions such as growth and differentiation in various cell types^15–17^. Recent evidence suggests that rigidity of the ECM surrounding cancer cells originating from lung or breast tissues mainly affects the expression of genes controlling proliferation and metastasis^18^; therefore, we studied the molecular mechanisms underlying the control of expression of these genes by ECM rigidity using non-small lung adenocarcinoma cell line, H1299. First, we cultured the cells on a collagen-coated soft (~0.5 kPa) or stiff (~40 kPa) polyacrylamide gel (PAG), which has the elasticity of normal or cancerous lung tissues^19^, respectively. Cells were found to be more spread and dispersed on the stiff matrix, indicating that these cells can sense and respond to the PAG stiffness (Fig. 1a). It has been reported that focal adhesion (FA) signalling is essential for cellular spreading on a substrate in response to sensing the matrix stiffness^20^; thus, we tested whether cell spreading on a stiff matrix is mediated by the canonical FA signalling (Fig. 1b,c). As previously reported for other cancer cell types^5, 21^, H1299 cells on the stiff matrix showed a fully developed vinculin-positive FA complex. Moreover, FA-related factors such as focal adhesion kinase (FAK) and extracellular signal-regulated kinase (ERK) were activated after matrix stiffening along with myosin light chain (MLC) phosphorylation. We showed that cell proliferation may be controlled by matrix stiffness (Fig. 1d), confirming that cellular growth is dependent on matrix rigidity as described in other reports^18, 22^.

To explore global gene expression patterns changed by matrix stiffness, we performed microarray analysis of samples obtained from H1299 cells cultured on a soft or stiff PAG. The increase in matrix stiffness upregulated various genes including proliferation-related genes as determined by gene ontology (GO) analysis (Fig. 1e); in particular, genes that are involved in EMT were enriched (associated with the GO term ‘cell proliferation’). In addition to increased expression of *DDR2*, we observed upregulation of *TWIST1*, *TWIST2*, and *ACTA2* (gene of α smooth muscle actin) on the stiff matrix, whereas *ZEB1*, *ZEB2*, and *CDH1* (gene of E-cadherin) among other EMT-related genes were slightly downregulated (Fig. 1f). Quantitative RT-PCR (qRT-PCR) results confirmed the microarray data, namely, that *DDR2* and *TWIST1* expression levels significantly and slightly increased, respectively, whereas *CDH1* expression significantly decreased (Fig. 1g).

### DDR2 is a key player in matrix stiffness–induced EMT

**Figure 2:**
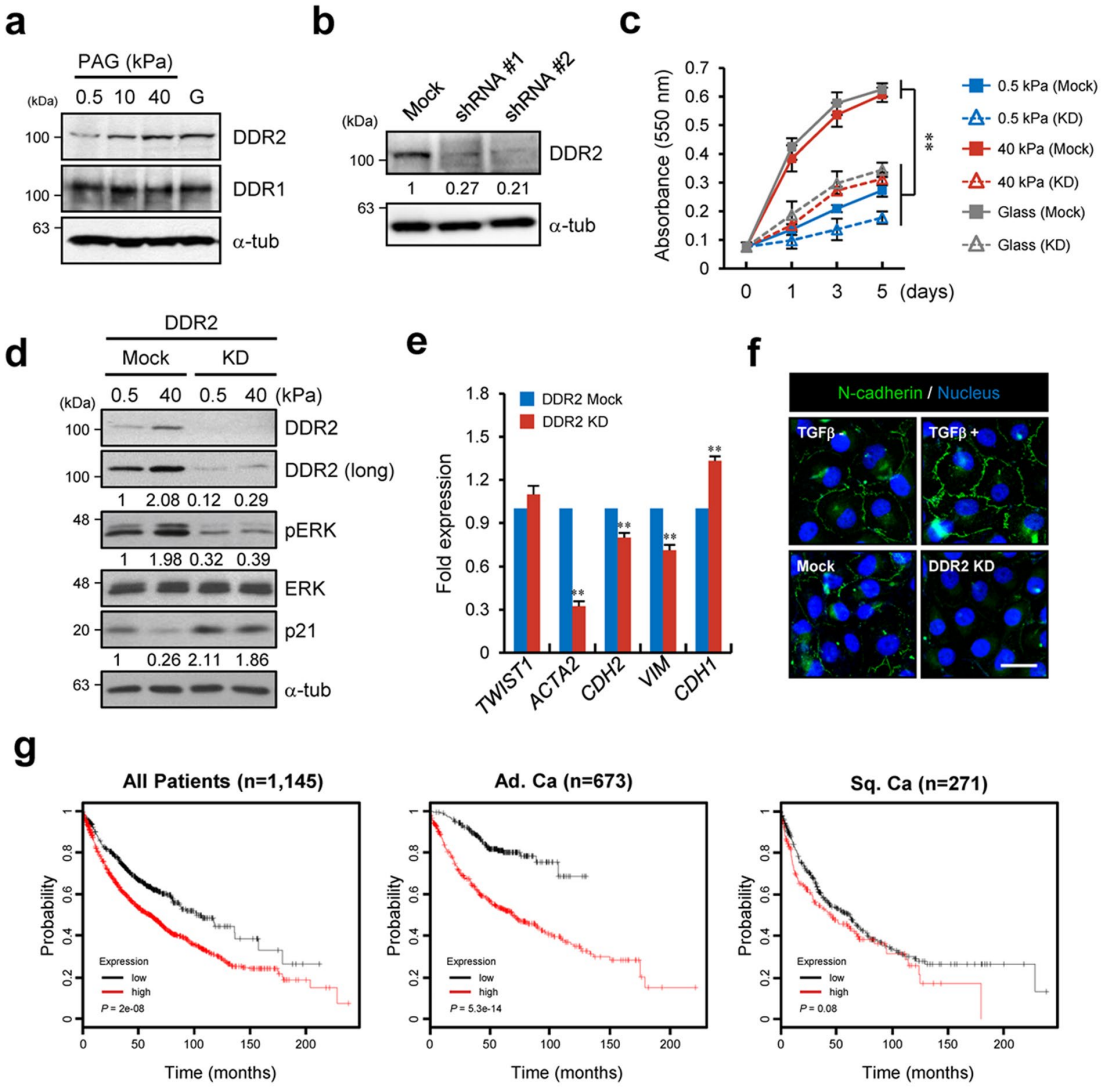
DDR2 is a key player in matrix stiffness–induced EMT. (**a**) Western blot analysis of DDR1 and DDR2 in H1299 cells incubated on a PAG with the indicated stiffness. (**b**) Western blot analysis of DDR2 in H1299 cells expressing mock or anti-DDR2 shRNA. (**c**) Quantification of proliferation of H1299 cells expressing mock or anti-DDR2 shRNA with indicated stiffness and duration. Data represent the mean of three independent experiments ± SEM. ^**^ *P* < 0.01. (**d**) Western blot analysis of DDR2, pERK, ERK, and p21 in H1299 cells expressing mock or anti-DDR2 shRNA under the indicated conditions. (**e**) qRT-PCR analysis of expression of *TWIST1*, *ACTA2*, *CDH2*, *VIM*, and *CDH1* in H1299 cells expressing mock or anti-DDR2 shRNA. Data represent the mean of three independent experiments ± SEM. ^**^ *P* < 0.01. (**f**) Immunostaining of N-cadherin and the nucleus in H1299 cells expressing mock or anti-DDR2 shRNA. Mock shRNA–expressing H1299 cells in the presence of 10 ng ml^−1^ TGF-β served as positive controls. (**g**) Kaplan–Meier curves of overall survival among patients with lung cancer, including lung adenocarcinoma and squamous cell lung carcinoma.

To ascertain whether DDR2 expression is regulated by matrix stiffness, we analysed DDR2 expression in H1299 cells incubated on PAGs of various stiffness or on glass. The results showed that DDR2 is specifically upregulated after matrix stiffening while expression of DDR1, another DDR isoform is independent on matrix stiffness in transcript and protein levels. (Fig. 2a and Supplementary Fig. 1a,b). Changing the composition of the ECM or serum in the culture medium did not affect the *DDR2* expression (Supplementary Fig. 1c,d). These results imply that ECM stiffness is a major regulator of *DDR2* expression but not *DDR1*.

Because DDR2 has been reported to regulate gene expression through activation of transcription factors^9, 23^, we determined whether DDR2 regulates EMT-related gene expression. After generation of DDR2 knockdown (KD) cell lines using short hairpin RNAs (shRNAs; Fig. 2b), we first tested the proliferative capacity on soft and stiff matrices because *DDR2* is categorised as a proliferation-related gene. As expected, DDR2 KD cells lost the capacity for proliferation on the stiff matrix (Fig. 2c). Examination of pERK and p21 levels in DDR2 KD cells indicated that DDR2 expression on the stiff matrix may modulate signalling and expression of cell cycle–regulatory proteins (Fig. 2d). We found that DDR2 KD cells showed weak expression of EMT-related genes such as *ACTA2*, *CDH2*, *VIM*, and *CDH1* (Fig. 2e). Immunocytochemical staining for N-cadherin revealed that N-cadherin expression was significantly decreased in DDR2 KD cells (Fig. 2f). Accordingly, these results suggested that DDR2 expression on a stiff matrix drives cancer cell proliferation and expression of a set of EMT-related genes. Furthermore, we evaluated the prognostic value of DDR2 using Kaplan–Meier Plotter^24^, an online meta-analysis tool for biomarker assessment (http://www.kmplot.com/analysis). According to this analysis, the expression level of DDR2 moderately correlates with overall survival of patients with lung cancer. Especially, it significantly correlates with a poor prognosis of adenocarcinoma, but DDR2 expression does not considerably correlate with squamous cell carcinoma (Fig. 2g). These results implied that DDR2 may act as an essential regulator in EMT related gene expression in stiff ECM environment.

### c-Myb and LEF1 are responsible for *DDR2* promoter activation

**Figure 3:**
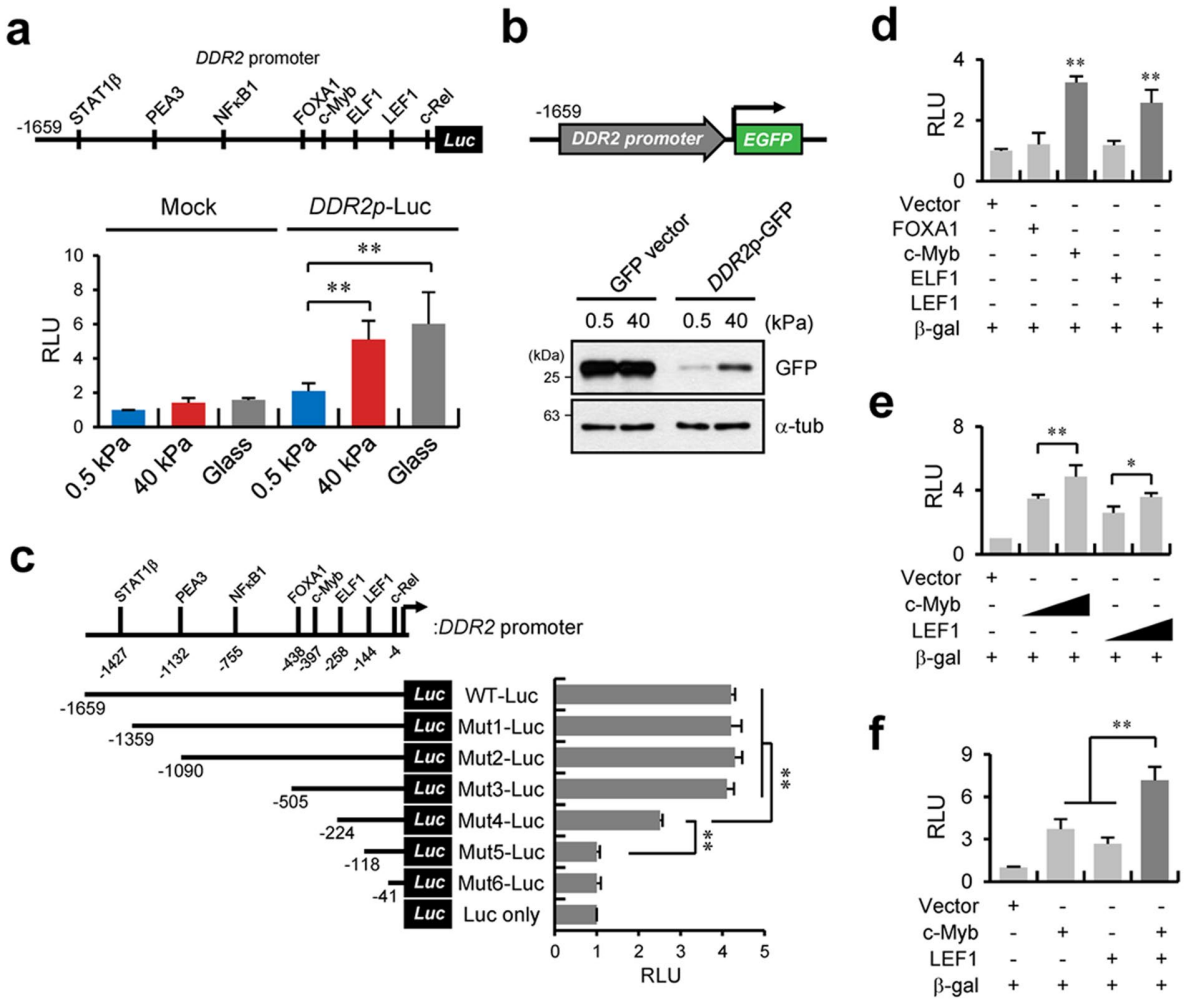
c-Myb and LEF1 are responsible for *DDR2* promoter activity. (**a**) Schematic representation of the *DDR2* promoter and possible relevant transcription factors (top). Relative expression of a mock or *DDR2* wild-type (WT) promoter-driven luciferase reporter in H1299 cells incubated on a PAG with the indicated stiffness or on glass (bottom). (**b**) Western blot analysis of green fluorescent protein (GFP) under the control of the *DDR2* WT promoter in H1299 cells incubated on a PAG with the indicated stiffness. (**c**) A schematic of the *DDR2* WT promoter and six truncated constructs in reporter vectors (top). Relative expression of *DDR2* WT or truncated promoter–driven luciferase reporters in H1299 cells incubated on a PAG with the indicated stiffness (bottom). (**d**) Relative expression of *DDR2* WT promoter–driven luciferase reporters in FOXA1-, c-Myb-, ELF1-, or LEF1-overexpressing H1299 cells (bottom). (**e**,**f**) Relative expression of *DDR2* WT promoter–driven luciferase reporters in c-Myb- or LEF1-overexpressing H1299 cells in a dose-dependent manner (**e**) and in co-overexpressing H1299 cells (**f**). Data in (**a**), (**c**), (**d**), (**e**) and (**f**) represent the mean of three independent experiments ± SEM. ^*^ *P* < 0.05,^**^ *P* < 0.01.

To understand the molecular mechanisms behind the matrix stiffness–dependent DDR2 expression, we analysed putative *cis-*acting elements in the *DDR2* promoter. Sequence analysis of the *DDR2* promoter predicted that 78 transcription factors possibly bind to the putative *cis-*acting elements in this promoter between positions −1659 and +27 (Supplementary Table 1). Among them, several *cis*-acting elements that have a high threshold value (>0.9) closely correlate with lung cancer progression (Supplementary Fig. 2). To determine which *cis*-acting elements are responsible for DDR2 expression, we constructed a *DDR2* promoter (WT-Luc) containing the above-mentioned *cis*-acting elements with a high threshold value. WT-Luc activity measurement showed that the *DDR2* promoter activity increased fourfold, just as under the conditions of a stiff matrix or glass (Fig. 3a). Expression of green fluorescent protein (GFP) under the *DDR2* promoter control confirmed the results on the luciferase-mediated DDR2 promoter activity. DDR2 promoter-GFP construct expression was strong on a stiff matrix, while CMV-GFP vector expression was independent of matrix stiffness (Fig. 3b). We next sought to determine which *cis-*acting elements are associated with the *DDR2* promoter activation; thus, reporter plasmids containing truncated fragments (Mut-1-Luc to Mut-6-Luc or Luc only) were generated. We showed that this promoter’s activity was reduced in cells transfected with Mut-4-Luc, and mostly downregulated in cells transfected with Mut-5-Luc (Fig. 3c). The Mut-4-Luc and Mut-5-Luc vectors lack 1435 and 1541 bp, respectively, at the 5′ end of the promoter. Therefore, our results suggest that the region between positions −505 and −118 containing the *cis*-acting element for the transcription factors FOXA1, c-Myb, ELF1, and LEF1 is crucial for *DDR2* promoter activation on a stiff matrix (Fig. 3c).

Next, we attempted to determine which transcription factors (among the four) are responsible for *DDR2* promoter activation under stiff-matrix conditions. A luciferase assay of the samples obtained from cells cotransfected with the *DDR2* WT-Luc construct and the GFP-tagged construct encoding FOXA1, c-Myb, ELF1, or LEF1 indicated that c-Myb and LEF1 coexpression resulted in a four- and threefold increase in *DDR2* WT-Luc activity, respectively (Fig. 3d). Dose-dependent overexpression of c-Myb and LEF1 confirmed that both transcription factors are specifically involved in the regulation of *DDR2* promoter activity (Fig. 3e). Furthermore, *DDR2* promoter activity was increased more than sevenfold during simultaneous expression of both transcription factors, as compared with cells transfected with each transcription factor individually (Fig. 3f). Collectively, these data suggest that c-Myb and LEF1 are responsible for the *DDR2* promoter activation on a stiff matrix.

### Increased cellular contractility on a stiff matrix is responsible for recruitment of c-Myb and LEF1 to the *DDR2* promoter

**Figure 4:**
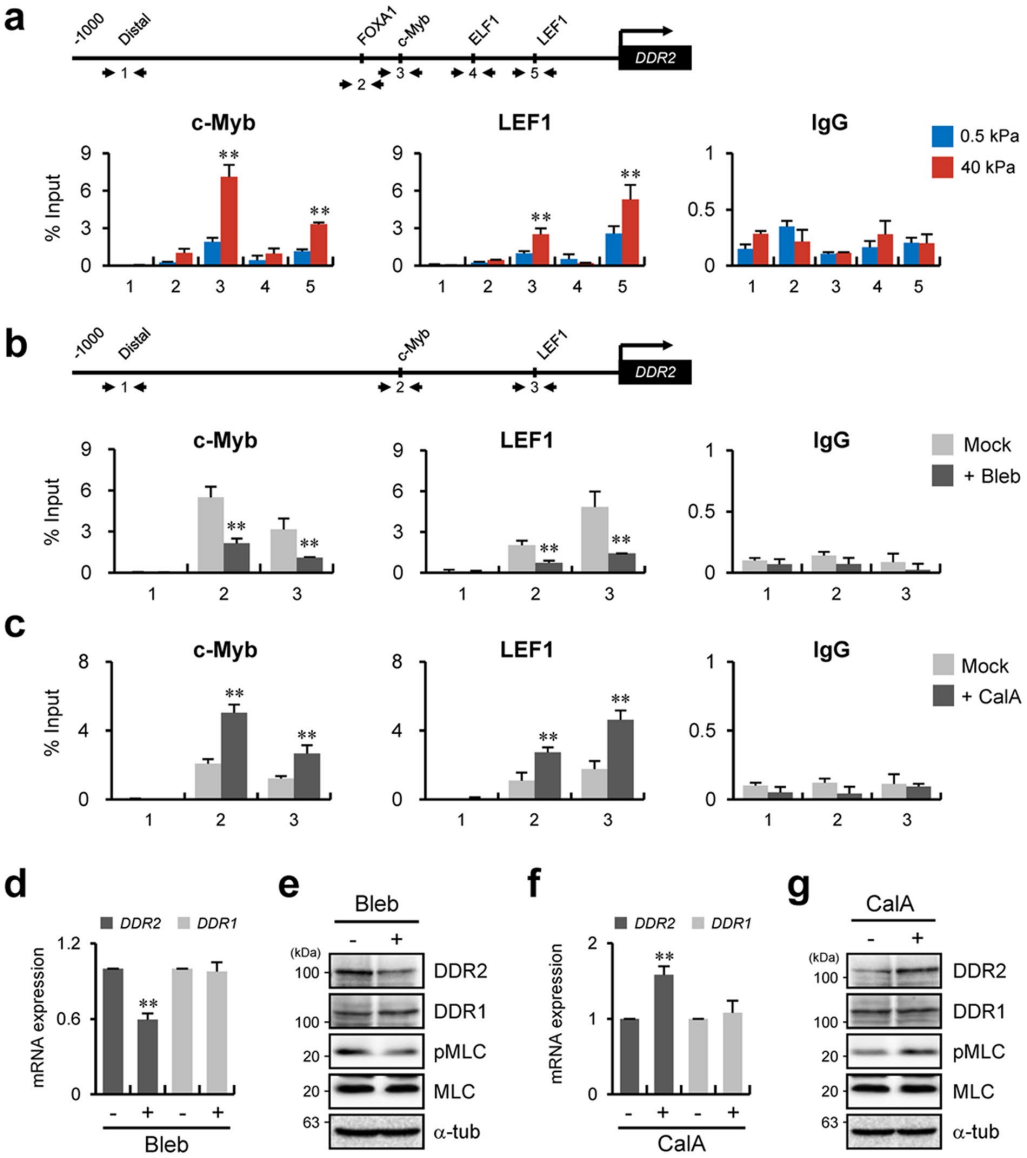
Increased cellular contractility on a stiff matrix is responsible for recruitment of c-Myb and LEF1 to the *DDR2* promoter. (**a**) Schematic depiction of the *DDR2* WT promoter with distal and putative binding sites for FOXA1, c-Myb, ELF1, and LEF1. Primer sets for chromatin immunoprecipitation (ChIP) analysis are indicated by the arrows in the scheme (top). ChIP analysis of the binding of c-Myb and LEF1 to the *DDR2* WT promoter in H1299 cells under the indicated conditions. qRT-PCR analysis with primers specific to one of five regions in the *DDR2* WT promoter as indicated in the scheme (bottom). (**b**,**d**,**e**) ChIP analysis of the binding of c-Myb and LEF1 to the *DDR2* WT promoter in H1299 cells in the absence or presence of 10 μM blebbistatin on a PAG with 40-kPa stiffness (**b**). qRT-PCR analysis of *DDR1* and *DDR2* (**d**) and western blot analysis of DDR1, DDR2, MLC, and pMLC **(e)** under the same conditions as in panel b. (**c**,**f**,**g**) ChIP analysis of the binding of c-Myb and LEF1 to the *DDR2* WT promoter in H1299 cells in the absence or presence of 2.5 nM calyculin A on a PAG with 0.5-kPa stiffness (**c**). qRT-PCR analysis of *DDR1* and *DDR2* (**f**) and western blot analysis of DDR1, DDR2, MLC, and pMLC (**g**) under the same conditions as in panel c. All data represent the mean of three independent experiments ± SEM. ^**^ *P* < 0.01.

To confirm that the recruitment of c-Myb and LEF1 to the *DDR2* promoter depends on the matrix stiffness in the cell, we performed a chromatin immunoprecipitation (ChIP) assay for c-Myb and LEF1 under the soft- and stiff-matrix conditions. Figure 4a showed that the recruitment of c-Myb and LEF1 to the *DDR2* promoter was significantly increased on a stiff matrix but not on soft matrix (Fig. 4a). The recruitment of c-Myb seemed to overlap with LEF1 recruitment and *vice versa*; this phenomenon is due to the interaction of these transcription factors^25^.

Because it was reported that a stiff substrate increases cellular contractility, whereas a soft substrate decreases the contractility^20^, it seemed possible that cellular contractility that is induced on a stiff matrix affects recruitment of c-Myb and LEF1 to the *DDR2* promoter. To test this hypothesis, we cultured H1299 cells on a stiff matrix with a myosin inhibitor, blebbistatin, to decrease cellular contractility. Under these conditions, recruitment of c-Myb and LEF1 to the *DDR2* promoter was significantly reduced (Fig. 4b). Conversely, enhancement of the cellular contractile activity by treatment with calyculin A (inhibitor of protein phosphatase 1) on a soft matrix increased the recruitment of the relevant transcription factors to the *DDR2* promoter (Fig. 4c). In support of these results, expression of DDR2 was significantly decreased by blebbistatin treatment on the stiff matrix (Fig. 4d,e), whereas the downregulation of DDR2 on the soft matrix was considerably attenuated after treatment with calyculin A (Fig. 4f,g). Collectively, these findings suggest that DDR2 expression is regulated by recruitment of c-Myb and LEF1 to the promoter in a cooperative manner, and this recruitment is controlled by cellular contractility induced by matrix rigidity.

### p300 regulates *DDR2* promoter activity via c-Myb acetylation

**Figure 5:**
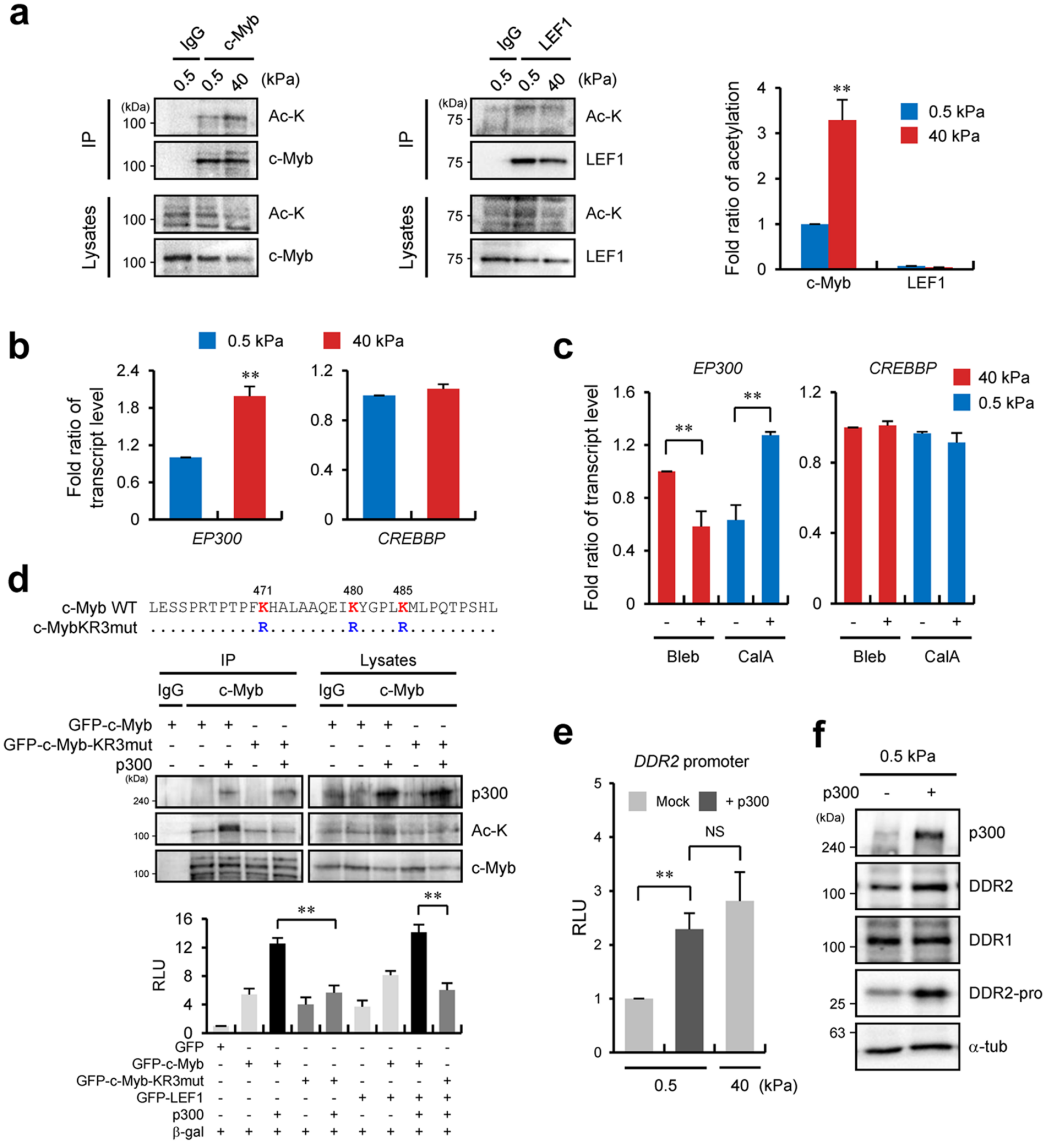
Histone acetyltransferase p300 regulates *DDR2* promoter activity via c-Myb acetylation. (**a**) A coimmunoprecipitation assay of lysates from c-Myb- or LEF1-overexpressing HEK 293 T cells using the indicated antibodies under the indicated conditions (left) and quantification of acetylation of c-Myb and LEF1 (right). Labels above and to the right of blots refer to antibodies used for the immunoprecipitation and immunoblotting, respectively. (**b**) qRT-PCR analysis of the expression of *EP300* and *CREBBP* in H1299 cells incubated on a 0.5- or 40-kPa PAG. (**c**) qRT-PCR analysis of expression of *EP300* in H1299 cells incubated on a 40-kPa PAG with blebbistatin (10 μM) or a 0.5-kPa PAG with calyculin A (2.5 nM). (**d**) A partial protein sequence of c-Myb. Conserved lysine residues at positions 471, 480, and 485 are highlighted in red. Arginine residues (blue) denote mutated amino acid residues (top). Coimmunoprecipitation analysis of lysates from c-Myb-, c-MybKR3mutant-, or p300-overexpressing HEK 293T cells using the indicated antibodies. Labels above and to the right of the blots refer to antibodies used for the immunoprecipitation and immunoblotting, respectively (middle). Relative expression of *DDR2* WT promoter–driven luciferase reporters in c-Myb-, c-MybKR3mut-, or p300-overexpressing H1299 cells as indicated (bottom). (**e**) Relative expression of *DDR2* WT promoter–driven luciferase reporters in mock or p300-overexpressing H1299 cells incubated on a PAG with 0.5- or 40-kPa stiffness. (**f**) Western blot analysis of p300, DDR1, and DDR2 in p300-overexpressing H1299 cells incubated on a PAG with 0.5-kPa stiffness. DDR2-pro refers to GFP under the control of the *DDR2* WT promoter. Data in (**a**), (**b**), (**c**) and (**e**) represent the mean of three independent experiments ± SEM. NS, non significant. ^**^ *P* < 0.01.

Post-translational modifications (PTMs), such as methylation and acetylation, of a transcription factor are required for regulation of gene expression^26^. Particularly, it has been reported that acetylation of c-Myb is necessary for target gene expression^27, 28^; accordingly, we tested whether increased matrix stiffness induces acetylation of c-Myb and LEF1. Immunoprecipitation analysis of c-Myb and LEF1 from cell lysates prepared under soft- or stiff-matrix conditions indicated that the extent of c-Myb acetylation significantly increased on the stiff matrix, whereas LEF1 was not affected (Fig. 5a). It has been reported that p300 (encoded by *EP300*) and CBP (encoded by *CREBBP*) HATs are involved in c-Myb acetylation^27, 28^. Therefore, we tested which HAT is responsible for c-Myb acetylation after matrix stiffening. Because we found *EP300* to be upregulated in the stiff-matrix condition in the microarray results, expression levels of *EP300* and *CREBBP* at different levels of matrix stiffness were examined next. qRT-PCR results showed that *EP300* expression increased in the stiff-matrix condition compared with the soft-matrix condition, while the expression of *CREBBP* was not changed at any stiffness (Fig. 5b). Next, we determined whether elevated expression of p300 on the stiff matrix also depends on the cellular contractility because cellular contractility increased the binding affinity of c-Myb for the *DDR2* promoter (Fig. 4b,c). qRT-PCR results revealed that treatment with blebbistatin on the stiff matrix inhibited the *EP300* expression, but this expression was markedly increased by treatment with calyculin A on the soft matrix. These findings indicated that increased expression of *EP300* in the stiff-matrix condition may be due to an increase in cellular contractility (Fig. 5c). Cotransfection of p300 with c-Myb increased the *DDR2* promoter activity significantly (10-fold), whereas coexpression of p300 with LEF1 did not affect the *DDR2* promoter activity. These data support the idea that the acetylation of c-Myb by p300 is involved in *DDR2* promoter activation (Supplementary Fig. 3).

Because it was reported that p300 can acetylate histone proteins, leading to chromatin remodelling and enhancement of transcription factor–DNA binding affinity^29^, we next tested whether acetylation of c-Myb by p300 directly participates in DDR2 expression. Because three lysine residues in c-Myb have been identified as acetylation sites of p300^27^, we generated a lysine-free c-Myb mutant (c-Myb-KR3mut) by substitution of lysines with arginines, and determined whether the c-Myb-KR3mut can activate the *DDR2* promoter. The c-Myb-KR3mut protein neither induced the acetylation by p300 (Fig. 5d, top) nor increased the *DDR2* promoter activity during coexpression with p300 (Fig. 5d, bottom), indicating that p300-mediated acetylation of c-Myb is crucial for *DDR2* promoter activation. In addition, we found that coexpression of c-Myb-KR3mut with LEF1 did not yield the additive effect (of c-Myb and LEF1) on *DDR2* promoter activity as compared with expression of wild-type c-Myb and LEF1 (strong additive effect on the promoter). These results imply that the cooperative effect of LEF1 and c-Myb on *DDR2* promoter activity is dependent on c-Myb acetylation status.

The above results indicate that downregulation of DDR2 on the soft matrix is due to an insufficient extent of c-Myb acetylation, which is a consequence of low expression of p300 in the soft-matrix condition. Thus, overexpression of p300 may increase DDR2 expression on the soft matrix. As expected, cells ectopically expressing p300 showed increased *DDR2* promoter activity even when these cells were cultured on the soft matrix (Fig. 5e). We next found that overexpression of p300 increased DDR2 expression and GFP synthesis driven by the modified luciferase vector shown in Fig. 3b (Fig. 5f). Collectively, these results suggested that higher expression of p300, c-Myb acetylation, and LEF1 are important for activation of DDR2 expression under stiff-matrix conditions.

### c-Myb is associated with lung cancer progression because of activation of DDR2 expression

**Figure 6:**
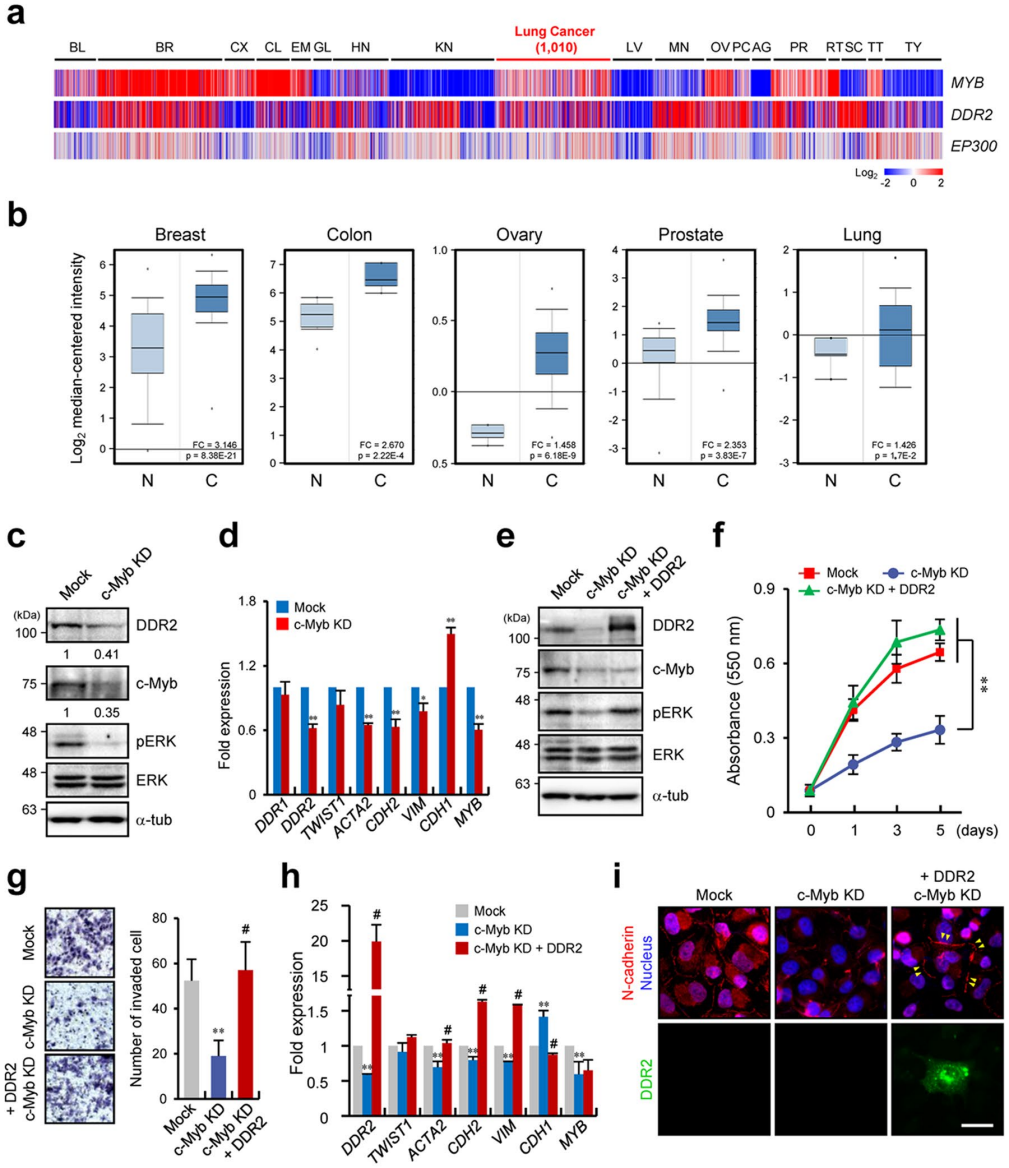
c-Myb is associated with lung cancer progression because of regulation of DDR2 expression. (**a**) Heat map representation of a TCGA pan-cancer RNA-Seq dataset from the Cancer Browser (Illumina HiSeq) showing the *MYB*, *DDR2*, and *EP300* expression levels in various cancer tissues. BL, bladder; BR, breast; CX, cervix; CL, colon; EM, endometrium; GL, glioma; HN, head and neck; KN, kidney; LV, liver; MN, melanoma; OV, ovary; PC, pancreas; AG, adrenal gland; PR, prostate; RT, rectum; SC, sarcoma; TT, testis; TY, thyroid. (**b**) An Oncomine boxed plot showing *MYB* expression levels in human breast, colon, ovary, prostate, and lung normal and cancer tissues. N, normal; C, cancer. (**c**) Western blot analysis of DDR2, c-Myb, phospho (p)-ERK, and ERK in H1299 cells expressing mock or anti–c-Myb shRNA. KD: c-Myb knockdown. (**d**) qRT-PCR analysis *of DDR1*. *DDR2*, *TWIST1*, *ACTA2*, *CDH2*, *VIM*, *CDH1*, and *MYB* under the conditions shown in panel c. Data represent the mean of three independent experiments ± SEM. ^*^ *P* < 0.05, ^**^ *P* < 0.01. (**e**) Western blot analysis of DDR2, c-Myb, pERK, and ERK in H1299 cells expressing mock or anti–c-Myb shRNA, or analysis of ectopically expressed DDR2 in H1299 cells expressing anti–c-Myb shRNA. (**f**,**g**) Quantification of proliferation (**f**) and invasion (**g**) shown by H1299 cells under the conditions shown in panel e. Data represent the mean of three independent experiments ± SEM. ^*^ *P* < 0.05,^**^ *P* < 0.01. (**h**) qRT-PCR analysis of *DDR2*, *TWIST1*, *ACTA2*, *CDH2*, *VIM*, *CDH1*, and *MYB* under the conditions shown in panel e. Data represent the mean of three independent of three experiments ± SEM.; ^*^ *P* < 0.05, ^**^ *P* < 0.01, ^#^ *P* < 0.01 as compared to c-Myb knockdown groups. (**i**) Immunostaining of N-cadherin and nuclei in H1299 cells under the conditions shown in panel e. The GFP signal indicates the cells ectopically expressing DDR2.

It has reported the importance of c-Myb on breast cancer progression^30, 31^, no report has yet been published regarding lung cancer. Since above results were obtained from the lung adenocarcinoma cell lines, we searched involvement of c-Myb in lung cancer progression using The Cancer Genome Atlas (TCGA) and Oncomine database. These analyses revealed that *MYB* expression (encoding the c-Myb) is increased in several cancers including breast, colon, and lung cancer as previously reported^31–33^, and *MYB* is overexpressed in lung cancer as compared with normal tissues (Fig. 6a,b).

To study the importance of the c-Myb–DDR2 axis for cancer progression including proliferation and invasion, we generated a c-Myb KD H1299 cell line and analysed gene expression and signalling, which were defective in DDR2 KD cells, as shown in Fig. 2. The c-Myb KD caused significant DDR2 downregulation and ERK activation (Fig. 6c). Furthermore, the EMT-related gene expression pattern in c-Myb KD cells was found to be similar to that presented in Fig. 2e (Fig. 6d). To confirm that lower expression of these genes in c-Myb KD cells is due to the DDR2 downregulation, we ectopically expressed DDR2 in c-Myb KD H1299 cells and quantified the proliferation, invasion, and patterns of gene expression. Forced expression of DDR2 induced pERK expression without any effect on c-Myb expression (Fig. 6e). Moreover, the decrease in cellular proliferation and invasion shown by c-Myb KD cells was significantly reversed by DDR2 overexpression (Fig. 6f,g and Supplementary Fig. 4). Furthermore, we investigated cell invasion by c-Myb KD cells under 3D conditions using spheroids^34^. The invasiveness of H1299 cells in the 3D matrix was significantly decreased by addition of GM6001, a general MMP inhibitor, and DDR2 and c-Myb KD cell lines yielded similar results (Supplementary Fig. 5). These data suggested that lower expression of DDR2 and c-Myb inhibits cell invasion under not only conventional 2D conditions but also 3D conditions.

In addition, we found that downregulated EMT markers such as *ACTA*, *CDH2*, and *VIM* were considerably upregulated, whereas *CDH1* was downregulated (Fig. 6h). Finally, immunostaining for N-cadherin indicated that the decrease in N-cadherin expression was reversed in DDR2-overexpressing cells (Fig. 6i). To confirm that these phenomena are not limited to H1299 cells, we generated a c-Myb KD in lung adenocarcinoma cell line A549, which is as metastatic as H1299 cells, but expresses wild-type p53. We found that expression of *DDR2* also increased on the stiff matrix but expression of *DDR1* was not changed. Also Increased *DDR2* expression on 40 kPa PAGs was decreased by the c-Myb KD (Supplementary Fig. 6a,b). In addition, proliferation and invasiveness of A549 cells were significantly decreased by the c-Myb KD (Supplementary Fig. 6c,d). Therefore, the downregulation of c-Myb resulted in a decrease of *DDR2* expression, leading to inhibition of cancer progression and downregulation of EMT marker genes.

It is known that integrin is a transmembrane receptor that mediates cellular responses to the rigidity of the ECM^35^. Because we found that cells are more spread on a stiff substrate, and canonical FA signalling is elevated depending on substrate stiffness, we tried to determine whether integrin is also involved in regulation of DDR2 expression and its induction depending on matrix stiffness. As a result of silencing of integrin β1, H1299 cells did not spread well on the stiff matrix (Supplementary Fig. 7a). Induction of ERK and MLC activity on the stiff matrix was also inhibited (Supplementary Fig. 7b). Besides, transcription levels of *DDR2* and *EP300* were markedly decreased by the KD of integrin β1 (Supplementary Fig. 7c,d), whereas transcription of the *MYB* gene was not changed significantly (Supplementary Fig. 7e). Besides regulation of DDR2 by c-Myb acetylation, these results may imply that integrin expression can be associated with the regulation of DDR2 expression by c-Myb and p300 depending on substrate stiffness.

## Discussion

**Figure 7:**
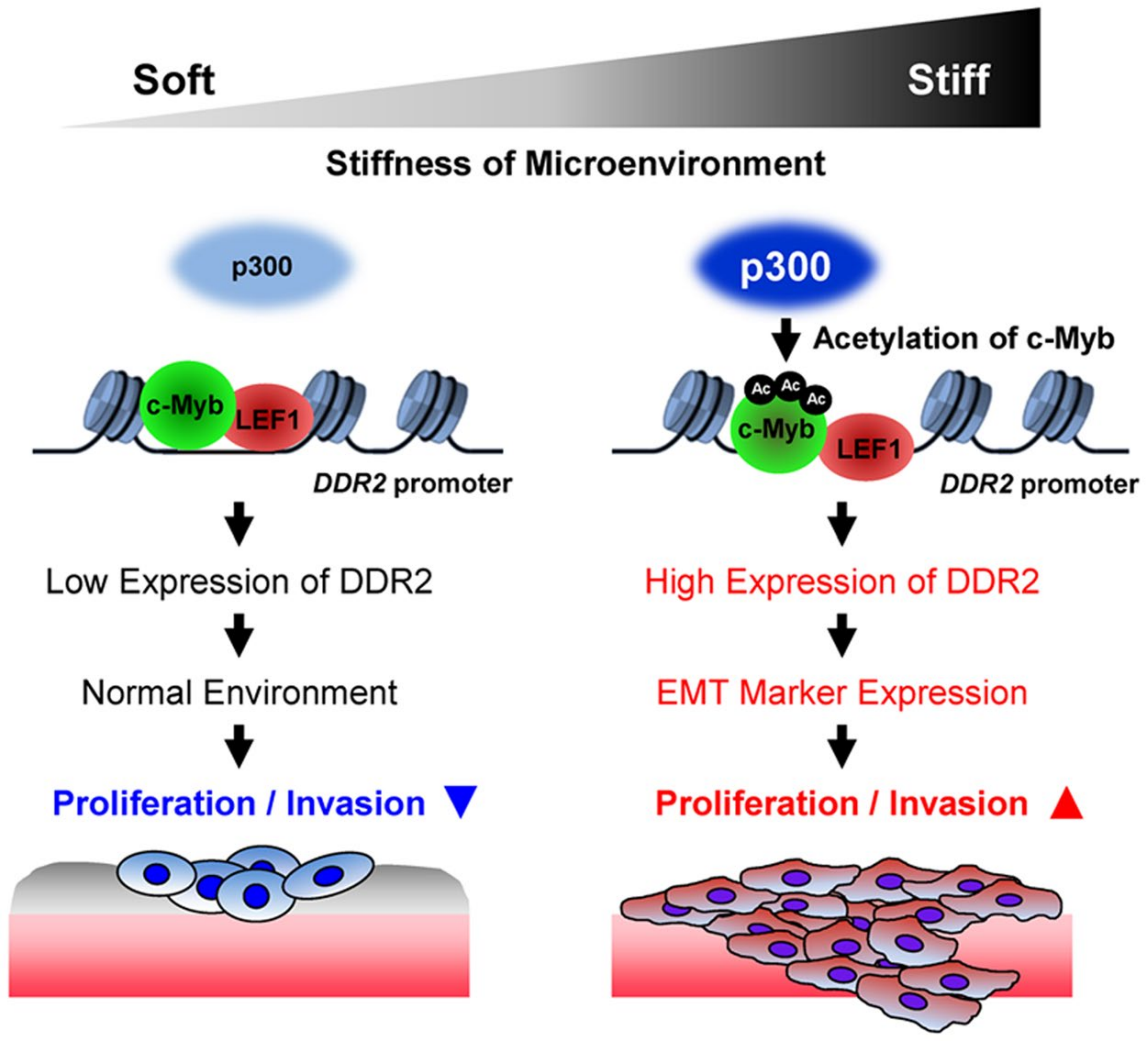
The proposed model of regulation of DDR2 expression by a combination of c-Myb, LEF1, and p300 in lung cancer. The overall model following from the results of this study suggestive of the mechanism behind the regulation of DDR2 expression via p300 and transcription factors c-Myb and LEF1 under the influence of ECM stiffness. On a soft matrix, physical binding between c-Myb or LEF1 and *DDR2* promoter is relatively weak. p300 expression is also weak, leading to lowered acetylation of c-Myb. Consequently, *DDR2* promoter is not very active under the soft-matrix conditions; therefore, cellular proliferation and invasiveness are low. On the other hand, direct binding of c-Myb, LEF1, and the *DDR2* promoter is significantly increased on a stiff matrix. p300 expression is also induced, resulting in increased acetylation of c-Myb, which enhances *DDR2* promoter activity. Thus, DDR2 expression is increased under the stiff-matrix conditions, thereby increasing cellular proliferation and invasiveness.

The increasing stiffness of cancer stroma is thought to provide a permissive environment for cancer development and EMT. Nonetheless, there are relatively few data on the mechanism behind the regulation of EMT progression by matrix stiffness. In this study, we demonstrated that DDR2 upregulation during cell culture on a stiff matrix may be crucial for expression of a set of EMT marker genes. DDR2 silencing significantly downregulated EMT marker genes, supporting the finding that DDR2 is a potent regulator of EMT marker gene expression in a stiff environment. Furthermore, we showed that *p300* upregulation on a stiff matrix led to c-Myb acetylation, promoting c-Myb and LEF1 recruitment to the *DDR2* promoter and leading to expression of DDR2 and EMT marker genes in the stiff stroma of a developing tumour (Fig. 7). DDR1 is reportedly involved in the TGF-β–induced EMT progression in lung adenocarcinoma and pancreatic cancer cells^8, 14^, but our microarray and qRT-PCR analyses showed that DDR1 expression is unchanged by matrix stiffness. Although no evidence of the relation between DDR2 expression and EMT has been reported, we showed that DDR2 – among EMT-related genes – is upregulated the most in the stiff-matrix condition (unlike DDR1). This finding implies that expression of EMT marker genes (associated with ECM stiffness) is related to DDR2. Thus, our results on mechanotranscriptional regulation of DDR2 expression by matrix stiffness seem to point to a new concept regarding how a mechanically permissive environment affects EMT and cancer progression.

We showed that DDR2 expression in a stiff environment requires c-Myb activity. c-Myb is reportedly required for progression of some cancers including leukaemia and breast and colon cancers^31–33, 36^. c-Myb upregulation in these cancers highly correlates with cancer development and metastasis^37^. Specifically, the role of c-Myb in the proliferation and differentiation of breast and colorectal cancer cells is studied intensively^30, 38, 39^. In contrast, studies on c-Myb’s physiological importance and function in lung cancer have been relatively infrequent although the expression of c-Myb in lung cancer has been reported^30, 37^. We confirmed c-Myb expression in lung tumour tissue by exploring TCGA and the Oncomine database (Fig. 6a,b). Furthermore, a c-Myb KD dramatically inhibited proliferation and invasiveness of H1299 and A549 lung cancer cell lines, indicating that c-Myb is likely essential for lung cancer progression.

DNA-binding affinity and transactivation capacity of c-Myb are reportedly regulated by PTMs such as phosphorylation and acetylation^27, 28, 40, 41^. Acetylation of lysine residues in the negative regulatory domain (NRD) of c-Myb by p300 or CBP increases the DNA-binding affinity and transactivation capacity of c-Myb^27, 28^. Meanwhile, phosphorylation of serine residues 11 and 12 by casein kinase II decreases DNA-binding affinity^42^, whereas phosphorylation of serine residue 528 in the NRD lowers transactivation capacity of c-Myb^43^. Because our results showed that p300 expression but not CBP expression in H1299 cells is elevated on a stiff matrix, p300 may participate in the regulation of c-Myb activity via acetylation in the stiff-matrix condition. Our results revealed that c-Myb transactivation capacity is increased by coexpression with p300, whereas lysine mutants of c-Myb show decreased transactivation capacity induced by p300. These data indicate that c-Myb acetylation by p300 may be the mechanism underlying the increase in DDR2 expression in a mechanically stiff environment.

Aside from regulation by acetylation, c-Myb is reported to cooperate with other transcription factors to activate target promoters. For example, the recombination-activating gene (*RAG*) promoter in a B-cell line contains a binding site for LEF1, c-Myb, and Pax5; these three transcription factors bind to the promoter region cooperatively to activate the *RAG* promoter^44^. In a study on leukaemia cell survival, c-Myb was found to bind (together with LEF1) to promoter regions of anti-apoptotic genes *Bcl2* and survivin^45^. Our results also indicated that c-Myb acts cooperatively with LEF1 to activate the *DDR2* promoter. Deletion of the c-Myb–binding site partially decreased the *DDR2* promoter activity, whereas simultaneous elimination of the LEF1-binding site completely suppressed the promoter activity. Additionally, promoter and ChIP analyses revealed that c-Myb and LEF1 are involved in the *DDR2* promoter activation via their synergistic DNA binding (Figs 3f and 4a). In contrast, coexpression of a c-Myb lysine mutant with LEF1 did not increase *DDR2* promoter activity (Fig. 5d), implying that the c-Myb acetylation by p300 prior to LEF1 binding is a prerequisite for *DDR2* promoter activation.

A growing body of evidence indicates that the ECM rigidity influences gene expression via the regulation of cellular localisation of transcriptional coactivators. Expression of α smooth muscle actin (α-SMA) is regulated by nuclear translocation of myocardin-related transcription factor (MRTF), thereby leading to activation of its partner transcription factor, serum response factor (SRF)^46, 47^. Treatment with a myosin inhibitor, blebbistatin, or a Rho kinase inhibitor, Y27632, blocks nuclear translocation of MRTF; this change decreases SRF-dependent gene expression^46, 47^. Similarly, YAP and/or TAZ as coactivators for transcription factor TEAD also inhibit its nuclear localisation after a decrease in the cell contractility by treatment with blebbistatin or Y27632^48–50^. In addition to the coactivator, a transcription factor itself was also reported to relocate into the nucleus after matrix stiffness increases^51–54^. Thus, nuclear relocation of a transcription factor and/or its coactivator seems to be a common phenomenon for gene expression induced by matrix stiffness. For c-Myb and LEF1, however, we did not observe nuclear relocation under the influence of matrix stiffness (Supplementary Fig. 8). Instead, the prominent change affecting these transcription factors on a stiff matrix is the acetylation of c-Myb, and we showed that the acetyl-free c-Myb mutant failed to increase *DDR2* promoter activity. Thus, these results suggest that the PTM of a transcription factor (in addition to regulation of nuclear localisation) can be important for the control of gene expression by matrix rigidity. Meanwhile, decreased actomyosin contractility reportedly leads to nuclear localisation of HDAC3, which catalyses deacetylation of histone H3 acetylated at lysine 9, thereby increasing chromatin compaction^55^. By contrast, HDAC3 cytoplasmic localisation causes chromatin loosening, thus triggering enhanced promoter activity of various genes, suggesting that epigenetic regulation at the genome level may be responsible for alteration of gene expression in response to matrix rigidity. Thus, identification of the molecular mechanism underlying PTMs of histone proteins and changes in chromatin structure may be worthwhile for further research into the effects of matrix mechanics on gene expression.

## Methods

### Cell culture and transfection

Non–small cell lung cancer H1299 and A549 cells were purchased from the Korean Cell Line Bank (Seoul, Korea). Human embryonic kidney 293T (HEK 293T) cells and lung normal epithelial HBE4 cells were acquired from the American Type Culture Collection (Manassas, VA, USA). HBE4 cells were cultured in the keratinocyte serum-free (KSF) medium (Gibco), H1299 and A549 cells were cultivated in RPMI 1640 (Invitrogen), and HEK 293T cells were cultured in Dulbecco’s modified Eagle’s medium (DMEM; Invitrogen) supplemented with 10% of foetal bovine serum (Hyclone). All cell cultures were maintained at 37 °C and 5% of CO_2_. Transient transfection procedures were performed using the TransFectin™ Lipid Reagent (Bio-Rad Laboratories Inc.) or a Neon^®^ electroporation device (Invitrogen).

### Plasmid construction

The pCMX-p300 plasmid was kindly provided by Professor Sangbeom Seo (Chung-Ang University, Korea). The construct of hemagglutinin-tagged *LEF1* provided by Professor Kwonseop Kim (Chonnam National University, Korea)^56^ and plasmids encoding *MYB*, *FOXA1*, and *ELF1* were prepared by a polymerase chain reaction (PCR) as reported previously^12^. The following primers were used: *MYB* forward 5′-CTAGCTAGCATGGCCCGAAGACCCCGG-3′, reverse 5′-CGGGGTACCATCATGACCAGCGTCCGGGC-3′; *LEF1* forward 5′-CTAGCTAGCATGCCCCAACTCTCCGGA-3′, reverse 5′-CGGGGTACCAGGATGTAGGCAGCTGTCAT-3′;*FOXA1* forward 5′-CTAGCTAGCATGTTAGGAACTGTGAAG-3′, reverse5′-CGGGGTACCGAGGAAGTGTTTAGGACGGG-3′; and *ELF1* forward 5′-CTAGCTAGCATGGCTGCTGTTGTCCAA-3′, reverse 5′-CGGGGTACCCAAAAAGAGTTGGGTTCCAG-3′. The DNA fragment was digested with NheI and KpnI and introduced into the pEGFP-N1 expression vector (Clontech). All the clones were verified by DNA sequencing.

The lentiviral shRNA constructs against puromycin, human *MYB* (TRCN0000040060, with the hairpin sequence 5′-CCGGGCTCCTAATGTCAACCGAGAACTCGAGTTCTCGGTTGACATTAGGAGCTTTTTG-3′ targeting the coding sequence of *MYB*) and *DDR2* #1 (TRCN0000121117, with the hairpin sequence 5′-CCGGCCCATGCCTATGCCACTCCATCTCGAGATGGAGTGGCATAGGCATGGGTTTTTG-3′ targeting the 3′ untranslated region of *DDR2* mRNA) and *DDR2* #2 (TRCN0000121121, with the hairpin sequence 5′-CCGGCCCTGGAGGTTCCATCATTTACTCGAGTAAATGATGGAACCTCCAGGGTTTTTG-3′ targeting the coding sequence of *DDR2*) were obtained from The RNAi Consortium (TRC) lentiviral shRNA library (Open Biosystems) and were cloned into the pLKO.1 vector (Addgene plasmid # 10878). Small interfering RNAs (siRNAs) targeting the coding sequence of integrin β1 (#1: 5′-CCCTCCAGATGACATAGAAA-3′) and the untranslated region of integrin β1 (#2: 5′-TAGGTAGCTTTAGGGCAATAT-3′) were synthesized by Genolution (Seoul, Korea).

The *DDR2* promoter region (positions −1,659 to +27 relative to the transcription initiation site) was amplified from H1299 cell genomic DNA using the following primers: forward, 5′-CTAGCTAGCTCTAGTTGTCTCTGGCAA-3′; reverse, 5′-CCGCTCGAGGAGAGAAACCTCACATTC-3′. The amplicon was digested with NheI and XhoI and ligated into the pGL4.12 vector (Promega). Site-directed mutagenesis of the three acetylation sites of *MYB*, replacing AAACA or TTAAA of the AAACATGCACTTGCAGCTCAAGAAATTAAA site with CGACA or TTCGA, and AAG of the TACGGTCCCCTGAAG site with CGG, was carried out using the PfuUltra High-Fidelity DNA Polymerase (Agilent).

### Generation of stable KD cell lines

These cell lines were generated using lentiviral plasmid vectors. Briefly, shRNA constructs were introduced by infection with lentiviruses. Concentrated viral supernatants were applied to target cells with 8 μg ml^−1^ polybrene. To create a stable cell line from H1299 cells, selection was performed in 1 μg ml^−1^ puromycin.

### Immunofluorescence microscopy

Cell preparations were fixed for 15 min with 3.7% (w/v) paraformaldehyde in phosphate-buffered saline (PBS), then blocked with 2% (w/v) bovine serum albumin (BSA) in PBS for 1 h. The samples were permeabilised for 10 min with 0.5% (v/v) Triton X-100 in PBS. The cells were then incubated with a primary antibody for 1 h, followed by incubation with the appropriate secondary antibody for 1 h at room temperature. The samples were analysed using an Eclipse 80i fluorescence microscope (Nikon) or an LSM 700 confocal microscope (Carl Zeiss). Images were acquired by means of a digital camera (DS-Qi1Mc, Nikon) and NIS-Elements image analysis software (Nikon). The images were processed and pseudo-coloured using Photoshop 11.0 (Adobe Inc.).

### PAG matrix preparation

The preparation of PAGs of various rigidity was performed as described elsewhere^17, 57^. We prepared PAGs ranging from 3% acrylamide/0.03% bis-acrylamide to 8% acrylamide/0.48% bis-acrylamide. PAGs were allowed to bind to 12- or 25-mm glass coverslips that had been pre-treated with 3-aminopropyltriethoxy-silane (Sigma-Aldrich). PAGs were then treated with 0.5 mg ml^−1^ sulfosuccinimidyl-6-[4′-azido-2′-nitrophenylamino]hexanoate (Thermo Fisher Scientific) and activated with ultraviolet light at 365 nm (2 × 10 min). The activated PAGs were coated with either 50 μg ml^−1^ neutralised collagen or a gelatine solution overnight at 4 °C. The gels were then thoroughly washed with PBS, equilibrated in DMEM or RPMI 1640, and used for luciferase assays, immunofluorescence microscopy, and RT-PCR assays.

### RNA isolation and RT-PCR

Total RNA was extracted from H1299 cells using the RNAiso Plus Reagent (Takara). RNA samples were spectrophotometrically quantified at 260 nm, and only samples that had an OD_260_/OD_280_ ratio between 1.9 and 2.0 were used. First-strand cDNA was prepared from 1 μg of RNA using oligo(dT) and M-MLV reverse transcriptase (Thermo Fisher Scientific). Amplification was conducted using Pfu DNA polymerase (Takara). RT-PCR and qRT-PCR were conducted using the Ex-Taq DNA Polymerase (Takara) and SYBR Premix Ex-Taq II (Tli RNase H Plus, Takara), respectively. The thermal cycling protocol consisted of an initial denaturation step at 94 °C for 5 min, followed by 30 amplification cycles (94 °C for 30 s, 57 °C for 30 s, and 72 °C for 30 s) for RT-PCR or an initial denaturation step at 94 °C for 5 min, followed by 50 amplification cycles (94 °C 10 s, 57 °C 10 s, and 72 °C 30 s) for qRT-PCR. All the qRT-PCR experiments were conducted independently three times in triplicate.

### Microarray analysis

This analysis was performed using H1299 cancer cells incubated on a soft (0.5 kPa) or stiff (40 kPa) PAG for 24 h by means of the Illumina HumanHT-12 v4 Expression BeadChip (Illumina), which includes a pool of unique bead types that correspond to 47,228 transcripts. Total RNA (0.55 μg) isolated from H1299 cells was reverse-transcribed and amplified, according to the protocols described in the Illumina Total Prep RNA Amplification Kit (Ambion). *In vitro* transcription was then carried out to generate cRNA (0.75 μg), which was hybridised onto each array and labelled with SA-Cy3 (FluoroLink^TM^ Cy^TM^3). The array was then scanned using the Illumina Bead Array Reader Confocal Scanner. Array data export processing and analysis were carried out in the Illumina GenomeStudio v2009.2 software (Gene Expression Module v1.5.4). For qRT-PCR, 1 μg of total RNA was used to synthesise cDNA. cDNA synthesis was primed using an oligo(dT) primer (Promega), and the quantified cDNA was used for analysis of a set of EMT markers: mRNAs of *DDR2*, *TWIST1*, *ACTA2*, *CDH2*, *VIM*, *CDH1*, *MYB*, and *GAPDH*. The following primers were used: *DDR2* forward 5′-CCACTATGCAGAGGCTGACA-3′, reverse 5′-CAGAGATGAACCTCCCCAAA-3′; *TWIST1* forward 5′-GAGTCCGCAGTCTTACGAG-3′, reverse 5′-GAGGACCTGGTAGAGGAAGT-3′; *ACTA2* forward 5′-TCCCTTGAGAAGAGTTACGA-3′, reverse 5′-CCCCTGATAGGACATTGTTA-3′; *CDH2* forward 5′-ATGAAGAAGGTGGAGGAGAA-3′, reverse 5′-TTAATGAAGTCCCCAATGTC-3′; *VIM* forward 5′-ACATTGAGATTGCCACCTAC-3′, reverse 5′-GTTTCGTTGATAACCTGTCC-3′; *CDH1* forward 5′-ATTCTGATTCTGCTGCTCTT-3′, reverse 5′-AACGTCGTTACGAGTCACTT-3′; *MYB* forward 5′-GCACCAGCATCAGAAGATGA-3′, reverse 5′-CATGACCAGCGTCCGGGC-3′; and *GAPDH* forward 5′-GAGTCAACGGATTTGGTCGT-3′, reverse 5′-TGTGGTCATGAGTCCTTCCA-3′.

The amplification reaction was conducted under the following conditions: 50 cycles of denaturation at 94 °C for 10 s, annealing at 57 °C for 10 s, and extension at 72 °C for 30 s. Dissociation curves were generated after each PCR run to ensure that a single product of the appropriate length was amplified. The mean threshold cycle (*C*_*T*_) and standard error were calculated from individual *C*_*T*_ values obtained from three replicates per stage. The normalised mean *C*_*T*_ was estimated as Δ*C*_*T*_ by subtracting the mean *C*_*T*_ of GAPDH from each value. ΔΔ*C*_*T*_ was calculated as the difference between the control Δ*C*_*T*_ and the values obtained for each sample. The fold change in gene expression, relative to the no-treatment control, was calculated as 2^−ΔΔCT^.

### Coimmunoprecipitation and western blotting

H1299 cells were transfected with one of the plasmids for 48 h. The resulting cell lysates were incubated with antibodies against c-Myb or LEF1, and next incubated with Protein A-agarose beads. Solubilised bead-bound materials were subjected to sodium dodecyl sulfate polyacrylamide gel electrophoresis (SDS-PAGE) and then transferred to polyvinylidene difluoride membranes (Millipore). The membranes were blocked, washed, and incubated with primary and secondary antibodies as indicated. Signals were developed using the enhanced chemiluminescence reagent (GE Healthcare), and band density was measured using a Quantity One^®^ system (Bio-Rad).

### Luciferase assays

HEK 293T or H1299 cells were transfected with 0.5 μg of pGL3 carrying the firefly luciferase reporter gene (Promega) and 0.5 μg of pCMV-β-galactosidase (Clontech), along with our expression vector by means of the TransFectin™ Lipid Reagent. After 48 h, the transfected cells were lysed with reporter lysis buffer (Promega), and the lysates were analysed using a GloMax^®^ Luminometer (Promega). Luciferase activity was normalised to β-galactosidase activity.

### The ChIP assay

H1299 cells were incubated on a soft or rigid matrix for 24 h. The cells were cross-linked with 1% formaldehyde, which was added to the medium with incubation for 10 min at room temperature, followed by incubation with 125 mM glycine for 5 min at room temperature. The cells were then lysed in SDS lysis buffer, after which the samples were sonicated and subjected to immunoprecipitation using various antibodies. The immunoprecipitates were eluted and cross-links were reversed; after that, the DNA fragments were purified using the PCR Purification Kit (Axygen). The precipitated DNA was analysed by qRT-PCR using *DDR2* promoter-specific primers. The following ChIP primers were used: *MYB* forward 5′-TGGTGGGAAGGGATTAAA-3′, reverse 5′-CTACCCCACACCTCATTTT-3′; *LEF1* forward 5′-GCGGTGTCTTTAAGTCTGTC-3′, reverse 5′-TATTGCACATTTGGTTTCAA-3′; *FOXA1* forward 5′-ATCCAGGACAGCAACAGAC-3′, reverse 5′- GCGGAGCTGACCTTTAG-3′; *ELF1* forward 5′-AAAAATGAGGTGTGGGGTAG-3′, reverse 5′-TACAGCAAAACCCATTCATA-3′; and distal region forward 5′-CAAAGGTAAGCTCGGTCTTC-3′, reverse 5′-CCAGGCTCCAGCCTTAAC-3′.

### 3D spheroid invasion assay

This assay was carried out as previously described with minor modifications^34^. Briefly, 2 × 10^4^ cells were seeded in suspension in agarose-coated plates. Then, the cells were coated with a type I collagen solution for 24 h. Next, a 1 mg ml^−1^ collagen mixture was poured into the wells and was incubated for polymerisation for 1 h. Spheroids were allowed to invade for 4 days, followed by fixation. Invasion was determined by measuring the circular area of the spheroid normalised to the original area of the spheroid. Fixed spheroids were stained for a proliferation marker with an anti-Ki-67 antibody (Cell signalling) and Alexa Fluor^®^ 488 conjugated phalloidin (Invitrogen).

### Quantification of cell proliferation with crystal violet

Cells (2 × 10^4^) were seeded in a 35-mm dish and incubated for various periods. At the end of incubation, the cells were washed three times with PBS and fixed with 3.7% paraformaldehyde for 15 min, and then were stained with 0.1% crystal violet for 30 min, after which the cells were washed and allowed to air dry. After that, 2% SDS was added to each sample and incubated for 20 min with gentle shaking. The extracted dye was quantified at 550 nm using an ELISA (Epoch, BioTeK).

### Statistical analysis

Differences between controls and treatment groups were evaluated by Student’s *t* test (between two groups) or by one-way analysis of variance (ANOVA; for three or more groups) in the GraphPad PRISM software. Data were expressed as mean ± standard error of the mean (SEM) of three independent experiments. Differences with *P* values less than 0.05 were considered statistically significant.

## Electronic supplementary material

Supplementary information
