# Supplementary material for: DDR2 controls the epithelial-mesenchymal-transition-related gene expression via c-Myb acetylation upon matrix stiffening
Source: Sci Rep. 2017 Jul 28;7:6847. doi: 10.1038/s41598-017-07126-7 (PMC5533734; doi:10.1038/s41598-017-07126-7)
Supplement: Supplementary file 1 — Supplementary information [file 41598_2017_7126_MOESM1_ESM.pdf]

DDR2 controls the epithelial-mesenchymal-transition-related gene expression via c-Myb acetylation upon matrix stiffening

Daehwan Kim[†], Eunae You[†], Jangho Jeong, Panseon Ko, Jung-Woong Kim and Sangmyung Rhee*

Department of Life Science, Chung-Ang University, Seoul 06974, Republic of Korea

[†] These two authors contributed equally to this work.

*To whom correspondence should be addressed:

Tel.: +82 2-820-5818

Fax: +82 2-825-5206

E-mail: sangmyung.rhee@cau.ac.kr

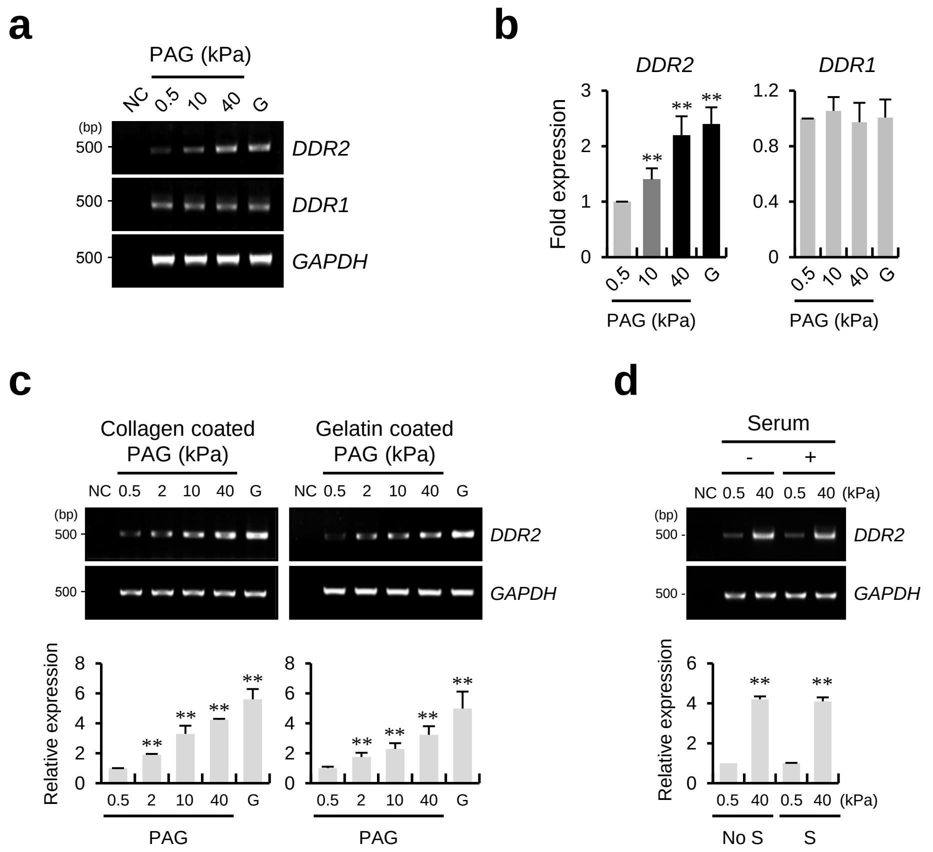

Supplementary Figure 1. Expression of *DDR2* transcripts is dependent on matrix rigidity. **(a,b)** RT-PCR **(a)** and qRT-PCR **(b)** analyses of expression levels of *DDR1*, *DDR2*, and *GAPDH* in H1299 cells incubated on a PAG of various stiffness or on glass (G). **(c)** RT-PCR analysis of the expression levels of *DDR2* and *GAPDH* in H1299 cells incubated on a PAG of various stiffness or glass coated with collagen or gelatine. **(d)** RT-PCR analysis of expression levels of *DDR2* and *GAPDH* in H1299 cells incubated on a PAG in the absence or presence of serum. Band densities were measured and normalised to *GAPDH* expression levels under the same conditions. NC, negative control. Data represent the mean of three independent experiments ± SEM. $^{**}P < 0.01$.

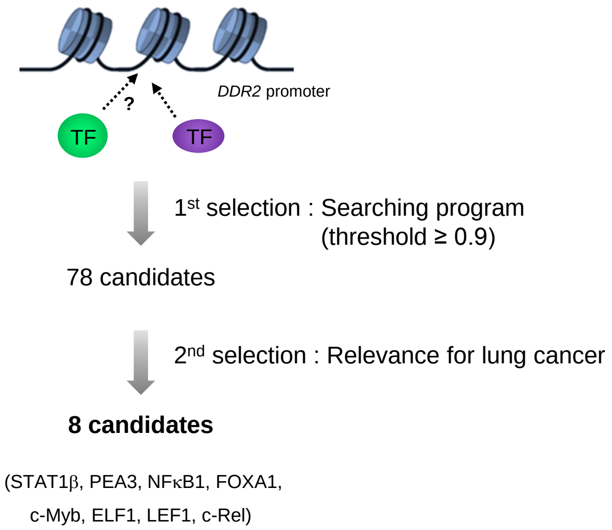

Supplementary Figure 2. The experimental scheme employed to analyse transcription factors that regulate *DDR2* promoter activity in lung cancer. Seventy-eight candidates are listed in Supplementary Table 1, and eight candidates (STAT1β, PEA3, NFκB1, FOXA1, c-Myb, ELF1, LEF1, and c-Rel) were eventually selected.

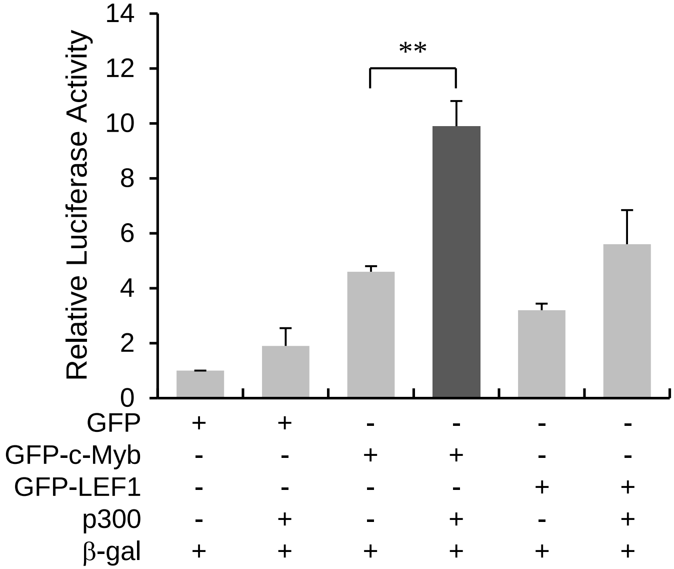

Supplementary Figure 3. Coexpression of c-Myb with p300 induces *DDR2* promoter activity. Relative expression of *DDR2* WT promoter–driven luciferase reporters in c-Myb-, LEF1-, or p300-overexpressing H1299 cells. Data represent the mean of three independent experiments ± SEM. $^{**}P < 0.01$.

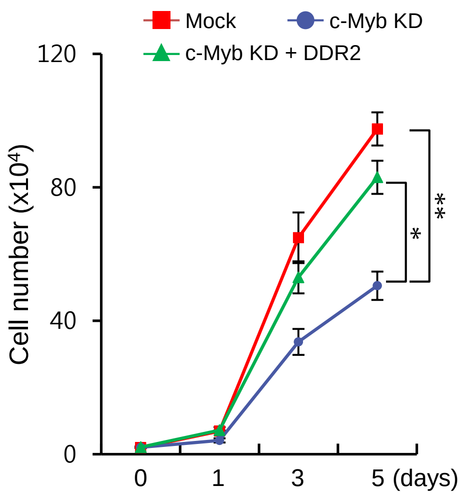

Supplementary Figure 4. Inhibition of proliferation in c-Myb knockdown H1299 cells is reversed by DDR2 overexpression. Quantification of proliferation of H1299 cells incubated on a stiff matrix for the indicated periods. The cells were trypsinized and counted. Data represent the mean of three independent experiments ± SEM. $^{*}P < 0.05$, $^{**}P < 0.01$.

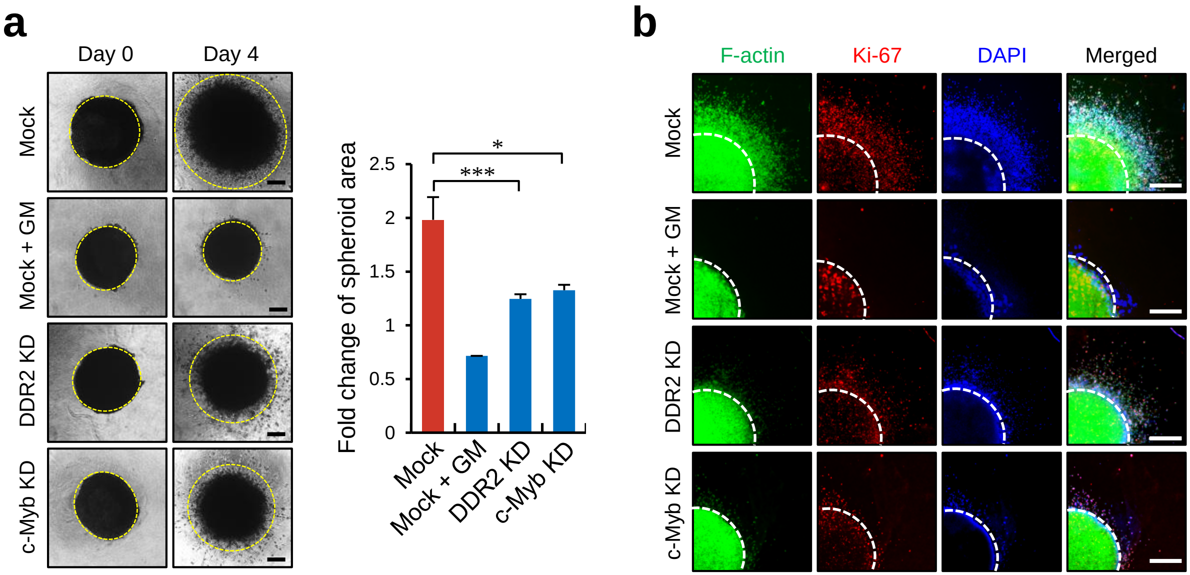

Supplementary Figure 5. A knockdown of c-Myb and DDR2 inhibits cancer invasion in a 3D environment. H1299 cells expressing mock (with or without 5 µM GM6001), anti-DDR2, or anti–c-Myb shRNA were incubated for 2 days to form spheroids as described in the *Methods* section. Collagen-embedded spheroids were further incubated for 4 days, fixed, and stained with phalloidin, an anti-Ki-67 antibody, and DAPI. The spheroids were examined by phase contrast microscopy **(a)** and fluorescence microscopy **(b)**. GM: GM6001. Scale bars: 200 µm (a) and 500 µm (b). Data represent the mean of three independent experiments ± SEM. $^{*}P < 0.05$, *** P< 0.001.

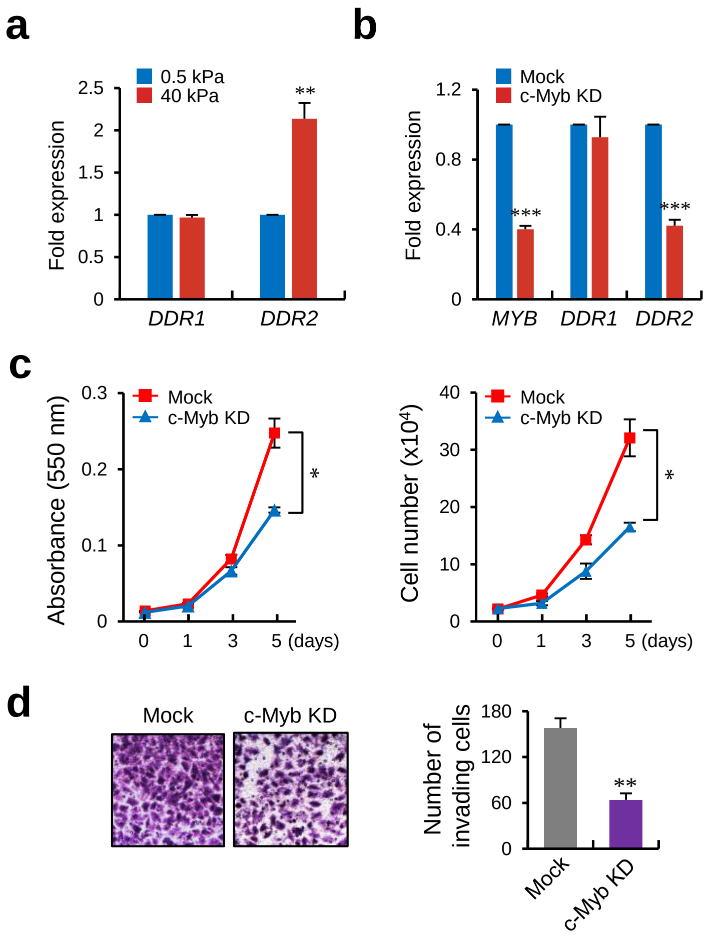

Supplementary Figure 6. The knockdown of c-Myb inhibits proliferation and invasiveness of A549 cells. **(a)** qRT-PCR analysis of *DDR1* and *DDR2* on 0.5- and 40-kPa PAGs in A549 cells. **(b) q**RT-PCR of *MYB*, *DDR1* and *DDR2* on 40 kPa PAGs in mock shRNA and c-Myb KD A549 cells. **(c)** Quantification of cell proliferation in mock or anti–c-Myb shRNA–expressing A549 cells. Graphs show absorbance of crystal violet (left) and the number of counted cells (right). **(d)** Quantification of invasion. Data represent the mean of three independent experiments ± SEM. $^{*}P< 0.05$, $^{**}P< 0.01$, $^{***}P< 0.005$.

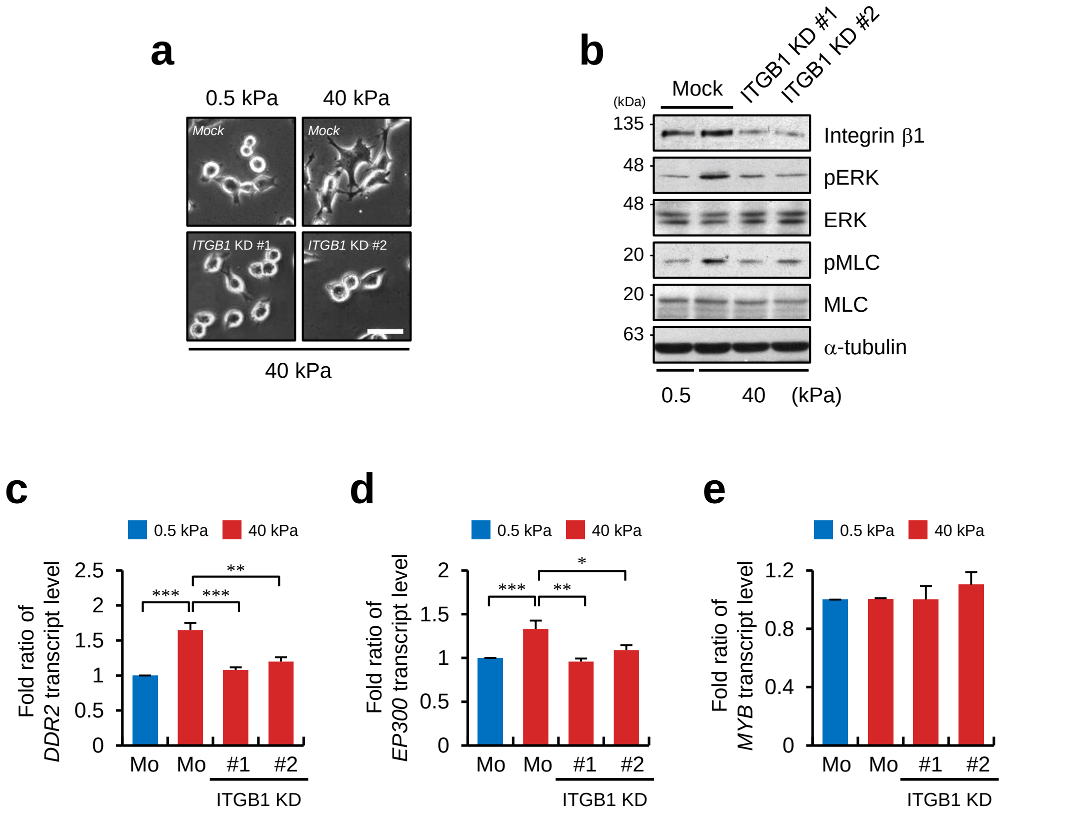

Supplementary Figure 7. Cellular contractility on a stiff matrix regulates the expression of *DDR2* and *EP300* via integrin β1. **(a)** A phase contrast image of H1299 cells transfected with mock siRNA or anti–integrin β1 siRNA (#1 and #2) on 0.5- and 40-kPa PAGs. Scale bar, 50 μm. **(b)** Western blot analysis of mock or anti–integrin β1 siRNA–transfected H1299 cells. **(c,d,e)** qRT-PCR analysis of expression of *DDR2* (c), *EP300* (d), and *MYB* (e) in mock and integrin β1 knockdown cells. qRT-PCR values were normalised to *GAPDH*. Mo: Mock. Data represent the mean of three independent experiments ± SEM. $^{*}P < 0.05$, $^{**}P < 0.01$, $^{***}P < 0.005$.

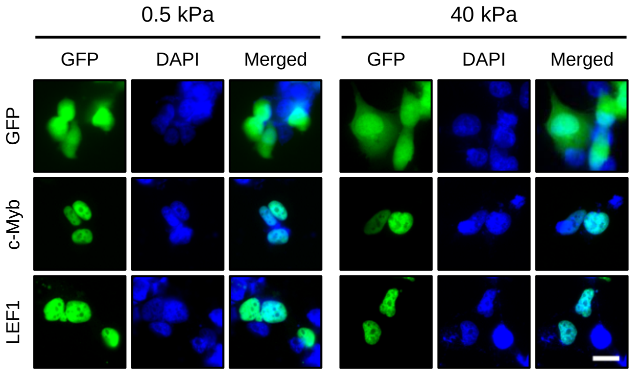

Supplementary Figure 8. Cellular localisation of c-Myb and LEF1 does not depend on matrix stiffness. Fluorescent images of H1299 cells transfected with plasmids encoding GFP, GFP-c-Myb, or GFP-LEF1 and incubated on a PAG. Nuclei were stained with DAPI. Scale bar, 10 µm.

Figure 1c

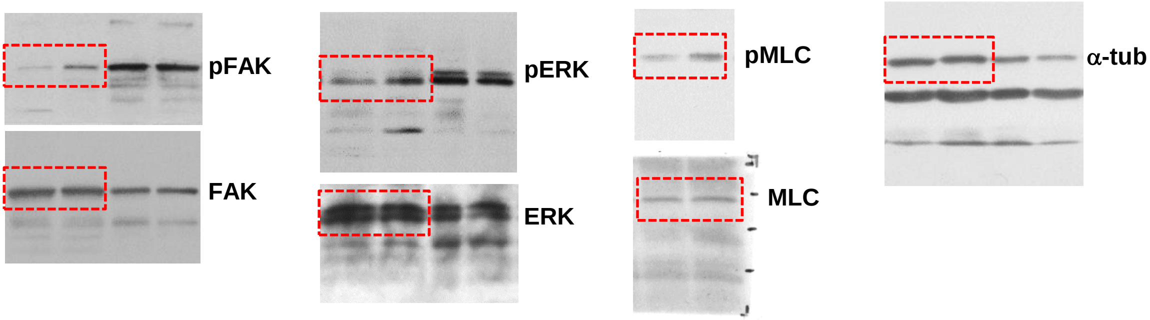

Figure 2a

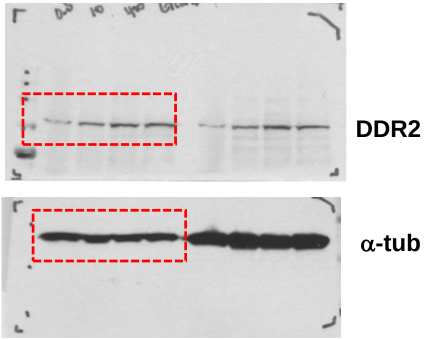

Figure 2b

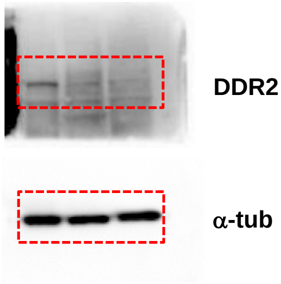

Figure 2d

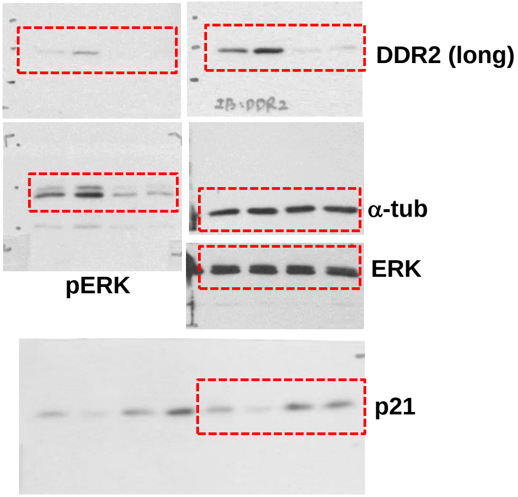

Figure 3b

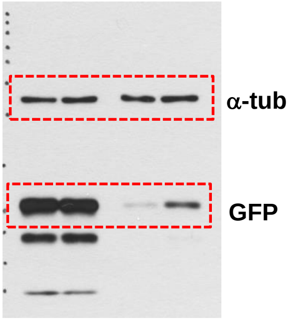

Figure 4e and 4g

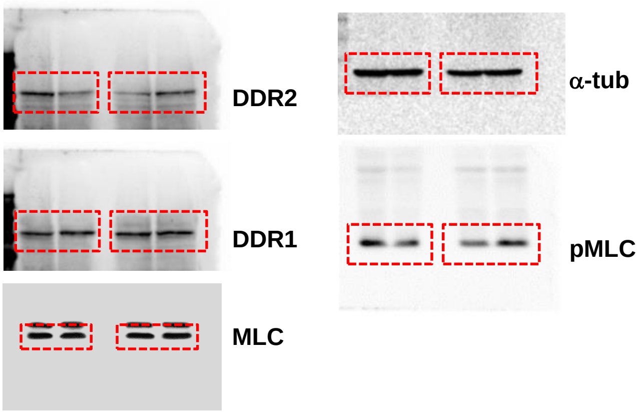

Figure 5d

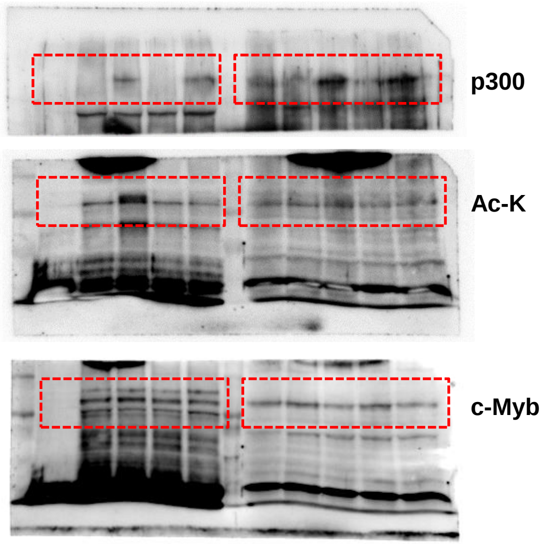

Figure 5f

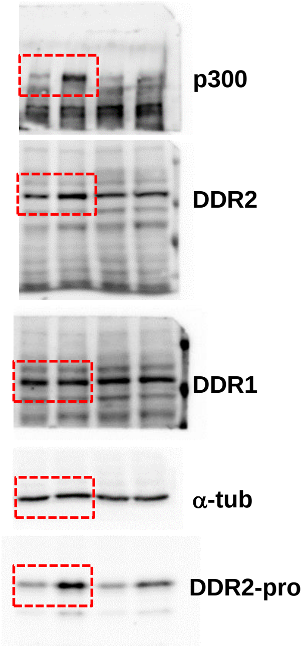

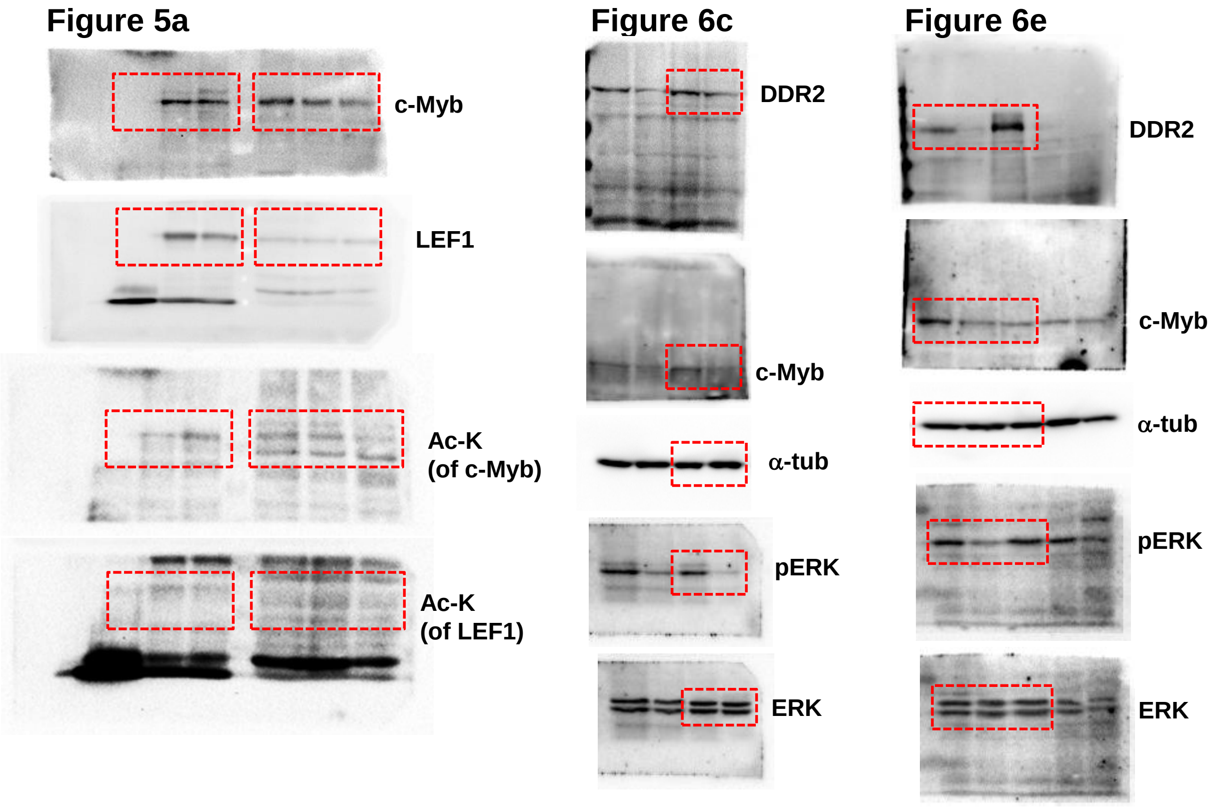

Supplementary Figure 9. Full length blots. Red dotted lines show the cropping locations.

Supplementary Table 1. Seventy-eight transcription factors that are predicted to bind to the *DDR2* promoter region.

No	Name	Description
1	FOXP3	Forkhead box P3
2	RXRα	Retinoid X receptor alpha
3	NR3C1	Nuclear receptor subfamily 3, group C, member 1 (glucocorticoid receptor)
4	RARβ	Retinoic acid receptor beta
5	GRα	Glucocorticoid receptor alpha
6	C/EBPα	CCAAT/enhancer binding protein alpha
7	C/EBPβ	CCAAT/enhancer binding protein beta
8	POU2F2	POU class 2 homeobox 2
9	c-Myb	Transcription factor c-Myb
10	FOXA1	Forkhead box A1
11	FOXA2	Forkhead box A2
12	XBP-1	X-box binding protein 1
13	TFIID	Transcription factor II D
14	c-Ets-1	V-ets erythroblastosis virus E26 oncogene homolog 1
15	c-Ets-2	V-ets erythroblastosis virus E26 oncogene homolog 2
16	IRF-1	Interferon regulatory factor 1
17	NFATC1	Nuclear factor of activated T-cells, cytoplasmic, calcineurin-dependent 1
18	NFATC2	Nuclear factor of activated T-cells, cytoplasmic, calcineurin-dependent 2
19	AP-2αA	Transcription factor AP-2-alpha A
20	p53	Tumor protein p53
21	AR	Androgen receptor
22	GATA-1	GATA binding protein 1 (globin transcription factor 1)
23	GATA-2	GATA binding protein 2 (globin transcription factor 2)
24	STAT1β	Signal transducer and activator of transcription 1beta
25	STAT4	Signal transducer and activator of transcription 4
26	STAT5A	Signal transducer and activator of transcription 5a
27	ATF3	Activating transcription factor 3
28	NFI/CTF	Nuclear factor I-CCAAT-binding transcription factor
29	NF-1	Neurofibromin 1
30	SRY	Sex-determining region Y
31	TCF-4E	Transcription factor 7 like 2, T cell specific, HMG box
32	VDR	Vitamin D (1,25- dihydroxyvitamin D3) receptor

33	PXR-1	Pregnane X receptor
34	c-Jun	Jun proto-oncogene
35	c-Fos	FBJ murine osteosarcoma viral oncogene homolog
36	HNF-1A	HNF1 homeobox A
37	HNF-1B	HNF1 homeobox B
38	YY1	YY1 transcription factor
39	POU2F1	POU class 2 homeobox 1
40	TEAD2	TEA domain family member 2
41	RBP-Jκ	Recombination signal binding protein for immunoglobulin kappa J region
42	PEA3	Phosphatidylinositol-4-phosphate 5-kinase and related FYVE finger-containing proteins signal transduction mechanisms
43	COUP-TF1	Nuclear receptor subfamily 2, group F, member 1
44	LEF-1	Lymphoid enhancer-binding factor 1
45	NF-κB1	Nuclear factor of kappa light polypeptide gene enhancer in B-cells
46	E2F-1	E2F transcription factor 1
47	SP1	Sp1 transcription factor
48	MAZ	MYC-associated zinc finger protein (purine-binding transcription factor)
49	TBP	TATA box binding protein
50	MEF-2A	Myocyte enhancer factor 2A
51	HOXD9	Homeobox D9
52	HOXD10	Homeobox D910
53	NF-Y	Nfy Histone-like transcription factor NFY protein family (IC)
54	Elk-1	Member of ETS oncogene family
55	EBF	Transcription factor COE1-like
56	Ik-1	IKAROS family zinc finger 1 (Ikaros)
57	ELF-1	E74-like factor 1 (ets domain transcription factor)
58	PU.1	SPI1 (PU 1) spleen focus forming virus (SFFV) proviral integration oncogene
59	WT1	Wilms tumor 1
60	Egr-3	Early growth response 3
61	AhR:Arnt	Aryl hydrocarbon receptor nuclear translocator
62	PPARα	Peroxisome proliferator-activated receptor alpha
63	TCF-4	Transcription factor 4
64	IRF-2	Interferon regulatory factor 2
65	USF2	Upstream transcription factor 2, c-fos interacting
66	Pax2	Paired box 2

67	Nkx-2.1	Homeobox protein Nkx-2.1
68	Sox-5	sex determining region Y-box 5
69	HSF1	Heat shock transcription factor 1
70	HSF2	Heat shock transcription factor 2
71	CP2	Transcription factor CP2
72	NF-E2	Nuclear factor, erythroid 2
73	MZF1	Myeloid zinc finger 1
74	Evi-1	Ecotropic viral integration site 1
75	c-Rel	V-rel avian reticuloendotheliosis viral oncogene homolog
76	NRF-2	Nf-E2 related factor 2
77	Lyf-1	Lymphoid transcription factor LyF-1
78	Pbx-1	Pre-B-cell leukemia homeobox 1
